# Measures of Bilingual Cognition – From Infancy to Adolescence

**DOI:** 10.5334/joc.184

**Published:** 2021-08-26

**Authors:** Lindsay Williams, Prasiddha Parthasarathy, Monika Molnar

**Affiliations:** 1 Department of Speech-Language Pathology, University of Toronto, CA; 2 DeGroote School of Medicine, McMaster University, CA; 3 Rehabilitation Sciences Institute, Faculty of Medicine, University of Toronto, CA

**Keywords:** bilingualism, cognition, development, systematic review

## Abstract

An extensive literature exists regarding the effect of bilingualism on cognition in developing populations. However, the term ‘cognition’ is vague and applies to a large number of different abilities. We reviewed 60 publications examining cognition in simultaneous bilingual children to understand what aspects of cognition have been studied in this population and what tasks have been used, in addition to qualitatively assessing the results of bilingual/monolingual comparisons. Executive function was the most frequently assessed cognitive ability across all age groups, paralleling the adult bilingual literature, with memory flexibility and theory of mind also emerging as common targets within infant and preschool age groups. Results are discussed in light of developmental trajectories and assessment methodologies currently available for the cognitive abilities represented in this literature.

The consequences of bilingualism on cognition have long been a focus of research. Many investigations have centered on the question of whether bilingualism confers advantages in performance on cognitive measures. It has been suggested that bilinguals are better at performing certain cognitive tasks than monolinguals, due to cognitive processing differences necessitated by the demands of using two languages rather than one (Bialystok, 2015; Kroll & Bialystok, 2013). The cognitive processes that facilitate the use of two languages, such as inhibitory control and task switching, are thought to generalize to non-linguistic domains. However, when it comes to actually observing the bilingual cognitive advantage during non-linguistic tasks, there is still a lively debate, regarding not only whether or not such an advantage exists, but also what methods are appropriate for studying it (e.g., Bialystok, Craik, & Luk, 2012; Bialystok & Werker, 2017; Costa, Hernández, & Sebastián-Gallés, 2008; De Bruin, Treccani, & Della Sala, 2015; Duñabeitia, Hernández, Antón, Macizo, Estéves, Fuentes, & Carreiras, 2014; Engel de Abreu et al., 2012; Gunnerud, Ten Braak, Reikerås, Donolato, & Melby-Lervåg, 2020; Grundy, 2020; Kroll & Bialystok, 2013; Lehtonen, Soveri, Laine, Järvenpää, de Bruin, & Antfolk, 2018; Paap, Johnson, & Sawi, 2015; Poarch & Krott, 2019). As a result, the number of publications addressing cognition in bilinguals continues to grow.

Different facets of cognition are often discussed in this extensive literature. ‘Cognition’ is an inherently elusive term, and Flavell, Miller, and Miller (1993) make this point well when discussing the challenge of producing a clear definition:

Once embarked on this course of broadening and restructuring the domain beyond the classical *higher mental processes*, it is very difficult to decide where to stop. One is finally led to ask, what psychological processes can*not* be described as “cognitive” in some nontrivial sense, or do *not* implicate “cognition” to a significant degree? The answer is that mental processes habitually intrude themselves into virtually *all* human psychological processes and activities, and consequently there is really no principled, nonarbitrary place to stop. (p. 2)

Considering the ambiguity the term ‘cognition’ presents, synthesizing what specific skills different studies are referring to when they use it and the tasks used to index those skills is the next best option for defining cognition as it is studied within a particular population. The goal of this systematic review is to examine what ‘cognition’ most commonly refers to in research on simultaneous developmental bilingual populations by identifying what aspects of cognition have been studied and the experimental tasks that have been used.

In work on bilingual adults, the cognitive abilities most frequently tested are the components of *executive function* (EF), which is typically described within the ‘classical *higher mental processes’*. Though definitions vary, EF is generally considered to encompass a range of cognitive abilities that allow for flexible, goal-directed behaviour and top-down control of responses (Gunnerud et al., 2020; Miyake, Friedman, Emerson, Witzki, Howerter & Wager, 2000; Roebers, 2017). These abilities include inhibition, attention, shifting, monitoring, and working memory (WM; or ‘updating’). In adult participants, EF components are most commonly assessed using variants of the Attention Network Task (ANT, attentional control; e.g. Costa, Hernández, & Sebastián-Gallés, 2008); Stroop and Simon tasks (inhibition; e.g. Bialystok, Craik, Klein, & Viswanathan, 2004); the Flanker task (attentional control; e.g. Costa, Hernández, Costa-Faidella, & Sebastián-Gallés, 2009); as well as task-switching paradigms in which subjects must change how they respond depending on a rule that changes periodically (e.g. Prior & MacWhinney, 2010).

In developmental bilingual populations, determining what ‘cognition’ most commonly refers to is somewhat more complicated, partially due to the wider range of cognitive abilities measured over the course of development. By definition, the cognitive abilities of developmental populations are changing and growing in complexity from birth till adolescence, so there is more variation in the skills measured and tasks used across infants, toddlers, and children. In addition, it is not always clear when a given cognitive ability typically emerges during development. As reflected by behavioural measures, the development of EF, for example, does not seem to progress smoothly but rather in spurts, and certain EF components emerge earlier than others (Anderson, 2002; Best & Miller, 2010). Inhibitory control of attention is generally considered to make its first appearance around the end of the first year of life in typically developing infants and then shows a rapid rate of development in the first three years (Diamond, 2013; Roebers, 2017). Task switching ability and the capacity to successfully deal with interference, in contrast, do not emerge until between three and five years of age (Anderson, 2002; Roebers, 2017). Importantly, once children have reached an age at which they can attempt these types of behavioural tasks, their further development tends to occur rapidly to the extent that they may perform at ceiling on tasks they struggled with a year prior (Roebers, 2017). Moreover, the complexity of tasks that can be performed and the speed with which they are performed continues to increase into middle and late childhood (Anderson, 2002; Roebers, 2017). Thus, the tasks used to assess EF in developmental populations must vary in a systematic way in complexity and difficulty depending on age, if meaningful differences are to be observed at each developmental stage across different groups (i.e., monolinguals vs. bilinguals).

Another ability that undergoes conspicuous development in childhood is theory of mind (ToM), the ability to reason about the mental states of others in order to explain and predict their behaviour. ToM has most frequently been assessed using false belief reasoning tasks in which children must predict how people will behave when they hold false beliefs about reality (e.g., thinking a toy is in one box when the child knows it has secretly been moved to another). Three-year-olds generally fail on these tasks, whereas four- or five-year-olds can reliably attribute false beliefs to others (Saracho, 2014). There is some evidence to suggest that ToM can be observed in infants as young as 15 months when they are tested using nonverbal measures (e.g., anticipatory looking paradigms; Poulin-Dubois & Yott, 2018; Saracho, 2014). However, whether these findings represent true ToM abilities is currently debated (see Poulin-Dubois & Yott, 2018; Powell, Hobbs, Bardis, Carey, & Saxe, 2018).

Given that children do not always have the same cognitive abilities available to them as adults do, what we refer to as cognition in (bilingual) children may be different from what it tends to refer to in adults. By systematically reviewing the abilities tested and the measures most often used when talking about cognition in bilingual and monolingual children, we can gain traction on the question of what facets of cognition are being referred to in the bilingual/monolingual cognitive development literature.

A prior review by Takakuwa (2000) examined several studies that reported a bilingual advantage for cognitive development in order to determine what the term ‘cognitive development’ actually referred to. The specific abilities targeted in the reviewed studies included intelligence, ‘cognitive strategies’, ‘control of processing’, and metalinguistic awareness. After critically examining each study, the review concluded that the only ‘cognitive development’ for which bilingualism could truly be said to confer benefit was the development of metalinguistic awareness, the ability to reflect on and manipulate the structures of language. Indeed, bilinguals have been frequently observed to outperform monolinguals on tasks that measure metalinguistic awareness (Sanz, 2019). This may be because learning two languages from an early age enables bilingual children to develop a more explicit awareness of the fact that language is a symbolic system for communication, allowing bilingual children to reflect on and manipulate linguistic structures earlier than monolingual children.

Since Takakuwa’s review, research on cognition in bilinguals has continued to accumulate rapidly. In their 2010 systematic review and meta-analysis, Adesope and colleagues examined the cognitive correlates of bilingualism in children and adults in 63 studies. The abilities covered included attentional control, problem-solving skills, creative and divergent thinking, cognitive flexibility, learning strategies, symbolic representation and abstract reasoning skills, metalinguistic awareness, metacognitive skills, and working memory. However, Adesope and colleagues (2010) did not explicitly discuss the differences between adult and developmental groups. Most recently, Gunnerud and colleagues (2020) conducted the first complete systematic review and meta-analysis of the pediatric bilingual advantage literature, focusing specifically on the bilingual advantage in EF. The EF components represented in the 100 publications reviewed were inhibition, switching, attention, monitoring, working memory, and planning. The authors also subdivided inhibition into ‘cold inhibition’, for tasks requiring the inhibition of an automatic or pre-potent response to neutral stimuli; ‘hot inhibition’, for tasks involving inhibiting a response related to obtaining a reward; and ‘attention inhibition’ for tasks requiring the participant to focus their attention on a target in the presence of distracting irrelevant stimuli. Tasks were assigned to these categories by the authors – the target abilities identified by the researchers in the original studies were not specified. The abilities with the largest number of effect sizes were cold inhibition and WM. Gunnerud and colleagues focused exclusively on differences in EF between bilinguals and monolinguals, but as Adesope et al. (2010) and Takakuwa (2000) have demonstrated, there are numerous other abilities that are tested in bilingual developmental populations. The present review will provide a broad exploration of the non-linguistic cognitive abilities as they are described and measured in *simultaneous bilingual* children, those who began to learn two languages before age 3.

## Objectives of Current Systematic Review

The primary goal of the current review is to disentangle what ‘cognition’ refers to in simultaneous bilingual developmental populations (0–18 years of age) by categorizing the various components of cognition that have been measured so far. This will likely include the components of EF, but we are also interested in surveying the literature on other cognitive abilities that have received less attention. We aim to understand how researchers talk about the different aspects of cognition they choose to measure, and to this end, we will categorize tasks according to the wording used by the authors to describe different target abilities when reporting our results. A second goal of this review is to identify the tasks used to measure these cognitive abilities, including classic tasks (e.g., Simon, Stroop) as well as less common or customized versions of tasks. A final objective is to qualitatively assess the effect of bilingualism on specific cognitive measures in children,^1^ which also requires consideration of some confounding factors, specifically socioeconomic status (SES), age of second language acquisition (AoA), and bilingual participants’ proficiency in their second language (Gunnerud et al., 2020; Kapa & Colombo, 2013; Luk, De Sa, & Bialystok, 2011; Naeem, Filippi, Periche-Tomas, Papageorgiou, & Bright, 2018).

In order to capture as much of the existing literature as possible and to understand what research typically refers to when examining cognition, our search strategy (described in the section titled ‘Search’ in Methods) was designed to include every paper that used the word ‘cognition’ in the title or abstract. This wide-reaching strategy was chosen to ensure we capture what have been considered to be measures of cognition. However, we applied some a priori guidelines for the types of cognitive skills we included. This was necessary in order to focus our review, as many tasks involve some cognitive component while relying more heavily on other domains (e.g., language, reading, use of heuristics). The focus of this review is the *non-linguistic* realms of cognition. For this reason, we did not include studies that investigated metalinguistic awareness, or include tasks that primarily measure linguistic responses/skills as an outcome; we also excluded studies examining quantitative skills and social communication skills (e.g., use of referential gestures). To answer our questions, we conducted a systemic review, to provide a more transparent, less biased, and comprehensive report of the currently available literature.

## Method

This systematic review was conducted according to the Preferred Reporting Items for Systematic Reviews and Meta-Analyses (PRISMA; Liberati, Altman, Tetzlaff, Mulrow, Gøtzsche, Ioannidis, Clarke, Devereaux, Kleijnen, & Moher, 2009; Moher, Liberati, Tetzlaff, Altman, The PRISMA Group, 2009). As the PRISMA guidelines were originally developed specifically for application to reviews that evaluate healthcare interventions, we have made modifications where appropriate given our research objectives. However, we adhered to the guidelines as closely as possible aside from these modifications. No ethical approval and/or consent was required for this study.

### Eligibility criteria

At the initial Title and Abstract screening stage, we included studies with the following characteristics:

- Participants were typically developing children between 0 and 18 years of age. Studies that recruited intellectually gifted children or children with developmental disabilities were excluded.
- Studies included an experimental group of bilingual participants and a control group of monolinguals, or bilinguals with lower proficiency in their second language (L2). Studies that tested bidialectal participants were excluded.
- Studies must have included a non-linguistic cognitive task as a main outcome measure. Studies were excluded if the tasks tested primarily linguistic or social psychological constructs (e.g. reading, personality measures), metalinguistic awareness, quantitative skills, or social communication skills. Additionally, cognitive measures that relied heavily on language (i.e. more than a word or short sentence) to give a response were excluded in order to minimize the possibility that performance was confounded by bilingual and monolingual children’s different experiences with language.
  As part of our critical appraisal of the studies, full texts were further assessed on the following two criteria important to research on bilingualism:
- Bilingual participants were simultaneous bilinguals (having acquired both of their languages before the age of 3), who had comparable proficiency in both languages (i.e. balanced bilinguals), as indicated by regular daily use of both languages or proficiency tests showing approximately equal proficiency in both. A relatively large body of literature exists on bilingual children’s cognitive development. This covers children with various age of acquisition (AoA) and proficiency levels. These factors have been shown to interact with the presence of cognitive advantages and also with language development in bilinguals (e.g., Luk et al., 2011; Perani et al., 1998; Bylund et al., 2019). For this reason, the current work focuses on simultaneous/early bilinguals (as a control for AoA and proficiency). Three years is a commonly accepted cut-off for simultaneous or early bilingualism within the literature (e.g., Dosi & Papadopoulou, 2019; Kapa & Colombo, 2013; Patterson, 2002), and although we acknowledge the arbitrariness of such a cut-off, we wished to remain consistent with other studies. It is also important to note that there is a body of literature that focuses on bilingual children with a later AoA for their second language (e.g., Adesope et al., 2010; Donnelly et al., 2019; Gunnerud et al., 2020), which could be the focus of a separate review. We excluded studies that did not provide sufficient information about bilinguals’ language background to determine AoA and proficiency levels, and those that tested exclusively second language learners. Studies that did not provide information about the languages spoken by participants were also deemed ineligible.
- Studies that did not report or control for SES were excluded.

We included articles in our review with the following report characteristics:

- Published, peer-reviewed articles reporting primary results. Grey literature, dissertations and master’s theses were not considered, because (i) we did not plan to conduct a meta-analyses and potential publication bias was not of concern, and (ii) to ensure we reviewed high-quality research (e.g., peer-reviewed).
- Written in English or French.
- No limits were imposed regarding the location or date of publication.

### Information sources

A comprehensive search was conducted using the following electronic databases: ERIC, Linguistic and Language Behavior Abstracts (LLBA), PsycInfo, Web of Science, and PubMed. A manual search of the reference lists of relevant past reviews of the bilingualism literature was also conducted (e.g., Adesope et al., 2010; Gunnerud et al., 2020). An updated manual search using Google Scholar was conducted March 8, 2021, and the most recent database search was run on July 2, 2021.

### Search

Search terms were defined by PP and MM in consultation with a librarian. All databases were searched using the following terms: cognit* AND pediatric* OR paediatric* OR child* OR adolescen* OR infan* OR preschool* OR toddler AND bilingual*. Where applicable (PsycInfo and PubMed), the limits ‘Human’, ‘All infants’, and ‘All child’ were used. To cover the widest range of articles, after running the search using the above strategy, the search was run a second time using ‘develop*’ in place of ‘pediatric* OR paediatric* OR child* OR adolescen* OR infan* OR preschool* OR toddler’.

### Study Selection

Study selection was performed in five stages. Duplicates were removed at the beginning of the screening process, and at each stage as new ones were discovered. Eligibility assessment was conducted by all the authors (LW, PP, & MM) and a research assistant (NJ and SR). In the first stage, one reviewer conducted a basic screen to remove articles that were clearly irrelevant to the review. In stages two, three, and four of the study selection process, titles, abstracts, and keywords were assessed and eligibility was indicated with ‘Yes’, ‘No’, or ‘Maybe’ by each reviewer. For each article deemed ineligible the reviewers indicated the reason based on the eligibility criteria. At stages three and four, articles for which at least one reviewer had said ‘Yes’ or ‘Maybe’ in the previous stage were assessed. After the fourth stage of screening, remaining disagreements between reviewers were resolved through discussion and by accessing the full-text articles. In the fifth stage, LW read in full all articles deemed eligible in previous stages and assessed them according to the eligibility criteria, yielding the final selection of studies included in the review. For a complete list of the articles that were excluded at the full-text stage with reasons for exclusion, see [*https://doi.org/10.5683/SP2/CBU7LL*].

### Data collection process

Data extraction was performed by LW in consultation with MM through a detailed inspection of each article.

#### Data items

The following data was extracted for each study: 1) participant age; 2) the type of control group used (e.g. monolingual vs. bilingual with lower L2 proficiency); 3) the characteristics of the bilingual participants (including descriptions related to socioeconomic status; language exposure and use; age of acquisition of the languages); 4) the languages spoken by bilingual participants; 5) the cognitive skills being assessed (as described by the original authors); 6) the cognitive measures used; 7) the results of comparisons between the control group and the bilingual group.

## Results and Interim Discussion

### Study selection

**Figure 1:**
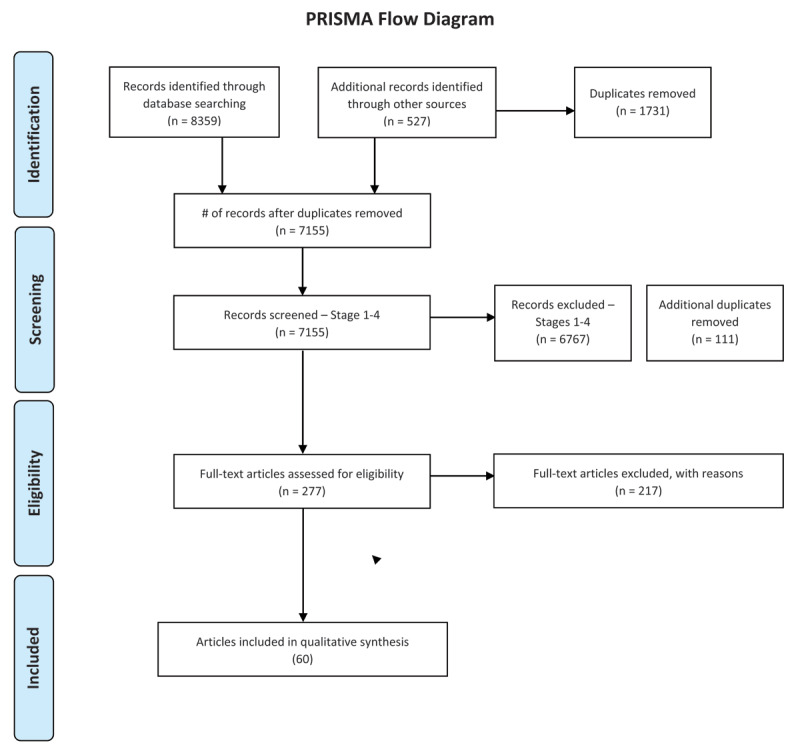
PRISMA Flow Diagram.

A flow diagram of the screening results at each stage is presented in ***Figure 1***. The search procedure yielded a total of 8,886 articles, and 277 records entered the full-text review. After these 277 articles were read in full and all excluded articles were recorded with the reasons for exclusion, 60^2^ articles remained for synthesis in the current review. The 60 articles included in the review reported results from a total of 71 individual studies/experiments.

### Study characteristics

The Appendix provides a detailed description of each publication included in the present review. We categorized the participants in the 60 publications selected for review into four age groups: infants (0–2; 6 years; Appendix, articles 1–14), preschool-aged children (2;6 – 6 years; Appendix, articles 15–38), school-aged children (6 – 12 years; Appendix, articles 39–57), and adolescents (13 - 18 years; Appendix, articles 58–60). The participants in the reviewed articles were recruited in 21 different countries, including Canada, the United States, Italy, the Netherlands, Singapore, Spain, Vietnam, Argentina, Romania, Israel, Iran, Germany, Australia, India, China, Luxembourg, South Africa, Greece, Belgium, Turkey, and Wales. However, the large majority of experiments recruited participants in Canada and the US. A total of 75 languages were represented in the bilingual groups tested, with homogeneous groups of bilinguals (40 across all studies) more common than heterogeneous groups (30). Within the subset of homogenous bilingual groups, English-Spanish, English-French, and Chinese (Cantonese or Mandarin)-English were the most frequent language pairings.

In the following sections, the primary cognitive outcome measures used in each age group are reported in two subsections for each group. In one subsection, the cognitive abilities measured and their frequency across experiments are presented, as well as the specific tasks used to measure them. Information about target abilities was identified from the introduction and methods sections of each article based on the author’s wording when describing the experiment(s), and we report them using the same terms used by the authors. In the second subsection for each age group, the frequency with which each cognitive task yielded differences between bilinguals and control groups is reported in table format. In a third subsection, the results for each age group are discussed individually, before the more detailed General Discussion section.

### Infant studies (0 to 2;6 years of age)

#### Target cognitive abilities and cognitive tasks used

As shown in the Appendix, we identified 14 articles (including 18 experiments in total) that tested infants. The target cognitive abilities measured by these experiments fell into the following categories: executive function, memory, perspective-taking, and basic information processing ability. The last two abilities were measured in one experiment each. Perspective-taking was tested in one experiment using a task in which children had to take the experimenter’s perspective in order to hand her the correct toy from two that were visible to them (Liberman, Woodward, Keysar, & Kinzler, 2017). Information processing was measured using a visual habituation task (Singh, Fu, Rahman, Hameed, Sanmugam, Agarwal, et al., 2015).

***Table 5*** shows the percentage of experiments that measured each target cognitive ability in each age group. Executive function (EF) was measured in a total of 11 experiments with infants, making it the most frequently measured ability in the infant group (Brito, Greaves, Leon-Santos, Fifer, & Noble, 2020; Brito, Grenell, & Barr, 2014; Comishen, Bialystok, & Adler, 2019, Study 1 & 2; Crivello, Kuzyk, Rodrigues, Friend, Zesiger, & Poulin-Dubois, 2016; Kalashnikova, Pejovic, & Carreiras, 2020; Kovacs & Mehler, 2009, Experiments 1–3; Poulin-Dubois, Blaye, Coutya, & Bialystok, 2011; Verhagen, de Bree, & Unsworth, 2020).

***Table 6*** lists all of the tasks used to measure EF across age groups along with the specific EF components they were used to measure. Two infant experiments employed a combination of conflict tasks, specifically the Multilocation task, Shape Stroop task, and Reverse Categorization task, and delay tasks, specifically Snack Delay and Gift Delay, as measures of inhibitory control, cognitive flexibility, working memory, and response suppression/control (Crivello et al., 2016; Poulin-Dubois et al., 2011). One experiment assessed inhibitory control and selective attention using the Spatial Conflict task and a visual search task respectively (Verhagen et al., 2020). This experiment also assessed attentional control and shifting via parental report using the Early Childhood Behavior Questionnaire (ECBQ). Five experiments measured selective attention and inhibitory control using a Visual Expectation Cueing Paradigm (VExCP; Comishen et al., 2019, Studies 1 and 2; Kovacs & Mehler, 2009, Experiments 1, 2, and 3). One additional experiment used the VExCP and defined their target ability as attentional control (Kalashnikova et al., 2020). Working memory was assessed in two experiments using the Spin the Pots and Hide the Pots tasks (Brito et al., 2014; Brito et al., 2020).

Memory was measured in seven experiments within six articles (Barr, Rusnak, Brito, & Nugent, 2020; Brito et al., 2020; Brito & Barr, 2012; Brito & Barr, 2014; Brito et al., 2014; Brito et al., 2015). All of these experiments used a Deferred Imitation Memory Generalization (DIMG) task in order to test infants’ memory flexibility (MF; the ability to generalize a previously learned response from one context to a novel, but similar one) and cued recall.

#### Differences between bilinguals and controls

For a detailed breakdown of the findings from each article in the infant age group see the Appendix. ***Table 1*** shows the differences found between infant bilingual and monolingual control groups based on task, with references to the Appendix number for each experiment.

**Table 1:** Infant Group Differences by Task.


COGNITIVE ABILITY	TASK (# OF EXPERIMENTS)	BILINGUAL ADVANTAGE EXPERIMENTS ^APPENDIX #^	MONOLINGUAL ADVANTAGE EXPERIMENTS ^APPENDIX #^	NO DIFFERENCES EXPERIMENTS ^APPENDIX #^

EF	ECBQ (1)			**1 ^14^**

	Gift Delay (2)			**2 ^7, 12^**

	Hide the Pots (1)			**1 ^4^**

	Multilocation (2)			**2 ^8, 12^**

	Reverse Categorization (2)			**2 ^8, 12^**

	Shape Stroop (2)	**1 ^12^**		**1 ^8^**

	Snack Delay (1)			**1 ^12^**

	Spatial Conflict Task (1)			**1 ^14^**

	Spin the Pots (1)			**1 ^5^**

	VExCP (6)	**4 ^7, 10^**		**2 ^7, 9^**

	Visual Search Task (1)			**1 ^14^**

Memory	DIMG (7)	**5 ^1, 2, 3, 4, 5, 6^**		**1 ^6^**

ToM	Visual Perspective-Taking**^a^** (1)	**1 ^11^**		

Info. Processing	Visual Habituation (1)	**1 ^13^**		


*Note*: Tasks are ordered by ability (EF, memory, ToM, information processing), then alphabetically. VExCP = Visual Expectation Cueing Paradigm; ECBQ = Early Childhood Behavior Questionnaire; DIMG = Deferred Imitation Memory Generalization task. ^a^ Visual Perspective-Taking yielded a bilingual advantage on an easy condition, but not a more difficult condition (Liberman et al., 2017).

#### Interim Discussion

The abilities that were tested most frequently in the infant group were executive function and memory flexibility (MF). MF was exclusively tested using the Deferred Imitation Memory Generalization (DIMG) task, which is well established for measuring infants’ ability to generalize a previously learned response from one context to another. EF was measured using a large variety of tasks, with the Visual Expectation Cueing Paradigm standing out as the most frequent.

While components of EF (inhibition, attention control) were commonly targeted abilities in this age group, it is not clear whether it is reasonable to expect differences in EF to appear in early infancy, as developmental studies lack clear evidence of EF during the first year of life (Ruff & Rothbart, 1996). One aspect of EF that has been convincingly observed in infants younger than 12 months is the ability to perform simple working memory (WM) tasks such as the A-not-B task (e.g., Diamond, 1985; Marcovitch & Zelazo, 2009), yet very few studies examined WM in the infant group. Notably, EF tasks overall rarely yielded differences between bilinguals and monolinguals in this age group, in contrast to the DIMG task for MF, which yielded a bilingual advantage in every experiment where it was used. The issue of measuring EF in infancy will be addressed in more detail in the General Discussion.

## Preschool-age studies (2;6 to 6 years of age)

### Target cognitive abilities and cognitive tasks used

Twenty-five articles (including a total of 29 experiments) tested preschool-aged children. One of these articles tested both preschool-aged and adolescent participants (Gathercole, Thomas, Viñas Guasch, Kennedy, Prys, Young, et al., 2016), and is also discussed in the adolescent section of our Results. As summarized in ***Table 5***, the target cognitive abilities measured by these experiments fell into the following categories: executive function, theory of mind, intelligence, general cognitive ability, and creativity.

EF was measured in 25 experiments within 21 articles, making it the most frequently tested cognitive ability in this age group (Aktan-Erciyes, 2020, Studies 1 and 2; Bain & Yu, 1980, Studies 1 and 2; Barac, Moreno, & Bialystok, 2016; Bialystok, 1999; Bialystok & Martin, 2004, Studies 1, 2, and 3; Carlson & Meltzoff, 2008; Crespo & Kaushanskaya, 2021; Diaz & Farrar, 2018a; Diaz & Farrar, 2018b; Dicataldo & Roch, 2020; Gathercole et al., 2016; Goldman, Negen, & Sarnecka, 2014; Haft et al., 2019; Leikin & Tovli, 2014; Mehrani & Zabihi, 2017; Namazi & Thordardottir, 2010; Nguyen & Astington, 2014; Tran, Arredondo, & Yoshida, 2015; Tran, Arredondo, & Yoshida, 2019; Yoshida, Tran, Benitez, & Kuwabara, 2010; Yoshida, Tran, Benitez, & Kuwabara, 2011).

As listed in ***Table 6***, four experiments used a child version of the Attention Network Task (ANT) to measure attentional control (Barac et al., 2016; Tran et al., 2015; Yoshida et al., 2010; Yoshida et al., 2011). Attention was additionally measured in three other experiments using the Simon task (‘controlled attention’; Namazi & Thordardottir, 2010), the Moving Word task (‘selective attention’; Bialystok, 1999), and the Conner’s Kiddie Continuous Performance test – Second Edition (K-CPT-2, ‘sustained attention’; Crespo & Kaushanskaya, 2021). Attentional fluctuations, represented by variability in response times on cognitive tasks, were measured in one experiment using the Tasks of Executive Control, a combination of *N*-back and go/no-go tasks (TEC; Haft et al., 2019).

Inhibitory control and response inhibition were assessed in 14 experiments using the child ANT (Carlson & Meltzoff, 2008), a visually cued recall task (Carlson & Meltzoff, 2008), the Kansas Reflection/Impulsivity Scale (KRISP; Carlson & Meltzoff, 2008), the Comprehensive Test of Nonverbal Intelligence (C-TONI; Carlson & Meltzoff, 2008), the Statue task (Carlson & Meltzoff, 2008), the Delay of Gratification task (Carlson & Meltzoff, 2008), Stroop and ‘Stroop-like’ (Day/Night, Happy/Sad) tasks (Diaz & Farrar, 2018a; Diaz & Farrar, 2018b; Dicataldo & Roch, 2020; Nguyen & Astington, 2014; Tran et al., 2019), Simon Says and Bear/Dragon Simon Says tasks (Carlson & Meltzoff, 2008; Diaz & Farrar, 2018a; Diaz & Farrar, 2018b; Tran et al., 2019), the Simon task (‘interference suppression’, Gathercole et al., 2016; Mehrani & Zabihi, 2017), the Gift Delay task (Barac et al., 2016; Carlson & Meltzoff, 2008; Tran et al., 2019), a Go/No-Go task (Barac et al., 2016), and a non-symbolic numerical discrimination task (Goldman et al., 2014). The Dimensional Change Card Sort task (DCCS) was also used to measure inhibition (Aktan-Erciyes, 2020, Studies 1 and 2; Bialystok, 1999; Bialystok & Martin, 2004, Studies 1, 2, and 3; Carlson & Meltzoff, 2008; Tran et al., 2019); this task was additionally used as a measure of cognitive flexibility in three experiments (Diaz & Farrar, 2018a; Diaz & Farrar, 2018b; Haft et al., 2019), shifting in five experiments (‘switching’, Aktan-Erciyes, 2020, Studies 1 and 2; ‘attention-shifting’, Dicataldo & Roch, 2020; Mehrani & Zabihi, 2017; ‘switching’, Tran et al., 2019), and selective attention and monitoring in one experiment (Tran et al., 2019). ‘Voluntary cognitive control’ was measured in two experiments, using a marble retrieval task similar to the Multilocation task, and a task similar to a Go/No-Go task in which children had to either squeeze a ball or withhold that response according to a cue (Bain & Yu, 1980, Studies 1 and 2).

Working memory (WM) was assessed in five experiments (Dicataldo & Roch, 2020; Leikin & Tovli, 2014; Mehrani & Zabihi, 2017; Namazi & Thordardottir, 2010; Nguyen & Astington, 2014). One experiment measured visual WM using a Pattern Recall task (Namazi & Thordardottir, 2010). Two experiments used a Listening Span task measuring verbal WM (Leikin & Tovli, 2014; Namazi & Thordardottir, 2010). Two experiments used both Forward and Backward Digit Span tasks (Dicataldo & Roch, 2020; Mehrani & Zabihi, 2017), and a final experiment used a Backward Word Span task (Nguyen & Astington, 2014).

ToM was assessed in seven experiments (Diaz & Farrar, 2018a; Diaz & Farrar, 2018b; Dicataldo & Roch, 2020; Goetz, 2003; Gordon, 2016; Kovacs, 2009; Nguyen & Astington, 2014). Of these, four experiments used exclusively false belief (FB) reasoning tasks (Diaz & Farrar, 2018a; Diaz & Farrar, 2018b; Dicataldo & Roch, 2020; Kovacs, 2009; Nguyen & Astington, 2014). These included the Unexpected Contents task, Unexpected/Change-in-Location task, Object Disappearance task, Appearance-Reality task (object property and object identity versions), and a modified false belief task (Kovacs, 2009). Another experiment used a level 2 perspective-taking task, in addition to FB reasoning tasks (Goetz, 2003). A sixth experiment assessed ToM using Diverse Desires, Diverse Beliefs, Knowledge Access, Belief-Emotion, Real-Apparent Emotions, and two FB tasks (Gordon, 2016).

Intelligence, general cognitive ability, and creativity were measured in one experiment each (intelligence: Darcy, 1946; ‘general nonverbal cognitive ability’: Gathercole et al., 2016; creativity: Leikin and Tovli, 2014). Darcy (1946) used the 1937 revision of the *Stanford-Binet Scale, Form L* to measure verbal intelligence and mental age, and the *Atkins Object-fitting Test, Form A* as a non-verbal measure of the same constructs. Leikin and Tovli (2014) tested children’s creativity using the Creating Equal Number (CEN) task. Gathercole and colleagues (2016) tested children using the McCarthy Scales of Children’s Abilities.

#### Differences between bilinguals and controls

Twenty-seven out of 29 experiments in the preschool group recruited monolinguals as the control group, and two experiments recruited children on a continuum of L2 exposure (Dicataldo & Roch, 2020; Haft et al., 2019). One experiment (Carlson & Meltzoff, 2008) included a group of immersion students with 6 months exposure to an L2 in addition to a monolingual control group. For a detailed breakdown of the findings from each article in the preschool age group see the Appendix. ***Table 2*** shows the differences found between preschool-aged bilingual and control groups by task, with references to the Appendix number for each experiment.

**Table 2:** Preschool-Age Group Differences by Task.


COGNITIVE ABILITY	TASK (# OF EXPERIMENTS)	BILINGUAL ADVANTAGE EXPERIMENTS ^APPENDIX #^	MONOLINGUAL ADVANTAGE EXPERIMENTS ^APPENDIX #^	NO DIFFERENCES EXPERIMENTS ^APPENDIX #^

EF	ANT (5)	**4 ^17, 35, 37, 38^**		**1 ^20^**

	C-TONI (1)			**1 ^20^**

	K-CPT-2 (1)			**1 ^21^**

	DCCS^a^ (13)	**8 ^18, 19, 20, 23, 32, 36^**		**5 ^15, 24, 25, 26^**

	Delay of Gratification (1)			**1 ^20^**

	FW Digit Span (2)			**2 ^25, 32^**

	BW Digit Span (2)			**2 ^25, 32^**

	Gift Delay (3)	**1 ^36^**		**2 ^17, 20^**

	Go/No-Go (1)	**1 ^17^**		

	KRISP (1)			**1 ^20^**

	Listening Span (2)	**1 ^31^**		**1 ^33^**

	Luria Stage 1 task (1)			**1 ^16^**

	Luria Stage 2 task (1)	**1 ^16^**		

	Moving Word (1)	**1 ^18^**		

	NSND (1)			**1 ^28^**

	Pattern Recall (1)			**1 ^33^**

	Simon (3)	**1 ^32^**		**2 ^33, 58^**

	Simon Says tasks (4)	**1 ^24^**		**3 ^20, 23, 36^**

	Statue task (1)			**1 ^20^**

	Stroop (+ Stroop-like; 5)	**1 ^36^**		**4 ^23, 24, 25, 34^**

	TEC (1)			**1 ^26^**

	Visually Cued Recall (1)	**1 ^20^**		

	BW Word Span (1)	**1 ^34^**		

ToM	Belief-Emotion (1)			**1 ^29^**

	Diverse Beliefs (1)			**1 ^29^**

	Diverse Desires (1)	**1 ^29^**		

	FB reasoning (6)			

	Appearance-Reality – Identity^a,b^ (3)	**3 ^24, 23, 27^**		

	Appearance-Reality – Property (1)	**1 ^24^**		

	Explicit FB (1)		**1 ^29^**	

	Modified FB task (1)	**1 ^30^**		

	Object disappearance (1)	**1 ^24^**		

	Unexpected location^a^ (5)	**4 ^23, 24, 30, 34^**		**1 ^27^**

	Unexpected contents^a,b^ (6)	**4 ^23, 24, 27, 34^**		**2 ^25, 29^**

	Knowledge Access (1)			**1 ^29^**

	Level 2 Perspective-taking^b^ (1)	**1 ^27^**		

	Real-Apparent Emotion (1)			**1 ^29^**

IQ	Atkins Object-fitting Test, Form A (1)	**1 ^22^**		

	Stanford Binet Scale, Form L (1)		**1 ^22^**	

Gen. Cog.	McCarthy Scales (1)			**1 ^58^**

Creativity	CEN (1)			**1 ^31^**


*Note*: Tasks are ordered by ability (EF, ToM, IQ, general cognitive ability, creativity), then alphabetically. ANT = Attention Network Task; C=TONI = Comprehensive Test of Nonverbal Intelligence; CEN = Creating Equal Number task; DCCS = Dimensional Change Card Sort task; K-CPT-2 = Conner’s Kiddie Continuous Performance Test – Second Edition; KRISP = Kansas Reflection/Impulsivity Scale; NSND = Non-Symbolic Numerical Discrimination task; TEC = Tasks of Executive Control. ^a^ In one study that followed a longitudinal design, the DCCS, Unexpected location, Unexpected contents, and Appearance-Reality, Object Identity tasks yielded a bilingual advantage at one time point but not another (Diaz & Farrar, 2018b).^b^ In one study, bilinguals showed an advantage for only one of two versions of each of the following tasks: Appearance-Reality: Object Identity, Level 2 Perspective-taking, and Unexpected Contents (Goetz, 2003).

#### Interim Discussion

Similar to the infant studies we reviewed, studies with preschool-aged participants also focused largely on EF. The focus on EF seems somewhat more justified in this age group given that these abilities are known to emerge and develop quickly between ages three and five (Anderson, 2002; Roebers, 2017). ***Table 2*** illustrates that the ANT and DCCS tasks were the most frequently used EF tasks among preschool children, with the DCCS in particular appearing in a large proportion of experiments, and these tasks yielded bilingual advantages in most experiments that used them.

The second most frequently measured ability in preschool children was theory of mind (ToM), which makes sense since our preschool age range includes the period in which ToM is generally considered to emerge (also between three and five years; Saracho, 2014). Consistent with existing trends in research on ToM in children of this age group, it was primarily measured using false belief tasks, the most common of which were the Unexpected Contents and Unexpected Location tasks, followed by Appearance-Reality tasks. These tasks have long been considered the “litmus test” for ToM in children (Poulin-Dubois & Yott, 2018), so their prominent use in the reviewed studies was to be expected. False belief tasks were consistently reported to yield advantages for bilinguals, as shown in ***Table 2***.

The pattern of results for EF and FB reasoning is consistent with research finding that FB reasoning relies on EF components like inhibitory control and shifting in order to hold two representations of reality in mind (the participant’s correct one and the other person’s incorrect one) and suppress one’s own representation in order to accurately predict the other person’s behaviour (Diaz & Farrar, 2018b). ToM and inhibitory control have been shown to be strongly correlated with one another (Carlson and Moses, 2001), so if preschool-aged bilinguals do indeed have an advantage in EF, a corresponding advantage for ToM should be expected to emerge as well.

## School-age studies (6 to 12 years of age)

### Target cognitive abilities and cognitive tasks used

The systematic review identified 20 articles (including a total of 23 experiments) that tested school-aged children. One of these articles (Kapa & Colombo, 2013) tested both school-aged and adolescent participants and is also discussed in the adolescent section of our Results. The target cognitive abilities measured by these experiments fell into the categories of executive function and intelligence.

***Table 5*** demonstrates that as with the infant and preschool groups, EF was the most frequently measured ability with school-age participants, with all 23 experiments measuring some component(s) of EF (Andreou et al., 2021; Antón, Duñabeitia, Estévez, Hernández, Castillo, Fuentes, et al., 2014; Bialystok & Viswanathan, 2009; Bosma, Hoekstra, Versloot, Arjen, & Blom, 2017; Cockcroft, 2016; Crespo et al., 2019; Czapka et al., 2020; de Abreu, 2011; de Abreu, Cruz-Santos, Tourinho, Martin, & Bialystok, 2012; Kapa & Colombo, 2013; Ladas, Carroll, & Vivas, 2015; Morales, Calvo, & Bialystok, 2013; Park, Ellis Weismer, & Kaushanskaya, 2018; Pino Escobar, Kalashnikova, & Escudero, 2018; Poarch, 2018; Poarch & van Hell, 2012; Poarch & Bialystok, 2015; Struys, Duyck, & Woumans, 2018; Tse & Altarriba, 2014; Vivas, Chrysochoou, Ladas, & Salvari, 2020). Attention was measured in 10 experiments using the ANT (attentional control: Antón et al., 2014; Kapa & Colombo, 2013; Ladas et al., 2015, Experiments 1 and 2; Poarch & van Hell, 2012, Experiment 2; Vivas et al., 2020), Simon task (attentional control: Poarch & van Hell, 2012, Experiment 1; Tse & Altarriba, 2014), and Sky Search task (selective attention; Bosma et al., 2017; de Abreu et al., 2012).

***Table 6*** shows that inhibition was assessed in 12 experiments using the DCCS (‘inhibitory control’, Crespo et al., 2019; Pino Escobar et al., 2018), Stroop task (Pino Escobar et al., 2018), Flanker task (Park et al., 2018; ‘conflict monitoring and inhibitory control’, Poarch, 2018; Poarch & Bialystok, 2015; ‘cognitive control’, Struys et al., 2018), the Bivalent Shape task (BST, ‘interference inhibition’, Czapka et al., 2020), the Go/No-Go task (‘response inhibition’, Czapka et al., 2020), Simon and Simon-like tasks (Morales et al., 2013, Study 1, ‘Pictures task’; ‘conflict monitoring and inhibitory control’, Poarch, 2018; Struys et al., 2018), the Frog Matrices task (Morales et al., 2013, Study 2), the Flanker task (‘interference suppression’: Bosma et al., 2017; de Abreu et al., 2012), the ANT (‘resistance to interference’, Vivas et al., 2020), and the ‘Faces task’ (‘inhibitory control and response suppression’, Bialystok & Viswanathan, 2009).

Shifting was assessed in four experiments, using the ‘Faces task’ (‘switching ability’, Bialystok & Viswanathan, 2009), DCCS (Crespo et al., 2019; Park et al., 2018), and the Simon Switching task (‘task switching’, Tse & Altarriba, 2014). Monitoring ability was assessed in two experiments using the DCCS (Crespo et al., 2019) and the ANT (Vivas et al., 2020).

Working memory was assessed in ten experiments (Andreou et al., 2021; Bosma et al., 2017; Cockcroft, 2016; Czapka et al., 2020; de Abreu, 2011; de Abreu et al., 2012; Morales et al., 2013, Study 1 & 2; Park et al., 2018; Tse & Altarriba, 2014). Four experiments measured verbal WM using Counting Recall, Forward and Backward Digit Span, and Non-Word Repetition tasks (Andreou et al., 2021; Bosma et al., 2017; Cockcroft, 2016; de Abreu, 2011). Visuospatial WM was tested in four experiments using Forward and Backward Dot Matrix, Odd-One-Out, Rotating Figure, and Frog Matrices (variant of Corsi Blocks) tasks (Andreou et al., 2021; Bosma et al., 2017; de Abreu et al., 2012; Morales et al., 2013, Study 2). WM was additionally assessed in five experiments using a Simon-like task (the Pictures task) with different levels of WM demand (Morales et al., 2013, Study 1), the Operation Span task (Tse & Altarriba, 2014), the Corsi Blocks task and the *N*-back task (‘updating’; Andreou et al., 2021; Czapka et al., 2020; Park et al., 2018).

Non-verbal intelligence was measured in two experiments using the Raven’s Colored Progressive Matrices test (Andreou et al., 2021; Cockcroft, 2016). The Raven’s test was also used by de Abreu et al. (2012) to measure ‘abstract reasoning ability’.

#### Differences between bilinguals and controls

Nineteen experiments in the school-aged group recruited monolinguals as controls, and four experiments had low-proficiency or sequential bilinguals as the control group (Bosma et al., 2017; Crespo et al., 2019; Poarch & van Hell, 2012, Experiment 2; Tse & Altarriba, 2014). For a detailed breakdown of the findings from each article in the school age group see the Appendix. ***Table 3*** shows the differences found between school-aged bi-/trilingual and control groups based on the task, with references to the Appendix number for each experiment.

**Table 3:** School-Age Group Differences by Task.


COGNITIVE ABILITY	TASK (# OF EXPERIMENTS)	BILINGUAL ADVANTAGE EXPERIMENTS ^APPENDIX #^	MONOLINGUAL ADVANTAGE EXPERIMENTS ^APPENDIX #^	NO DIFFERENCES EXPERIMENTS ^APPENDIX #^

EF	ANT (5)	**2 ^54, 59^**		**3 ^46, 40, 57^**

	BST (1)	**1 ^45^**		

	Corsi Blocks (1)			**1 ^50^**

	Counting Recall (2)			**2 ^43, 46^**

	Day/Night Stroop (1)			**1 ^51^**

	DCCS (3)	**1 ^50^**		**2 ^44, 51^**

	FW Digit Span (2)			**2 ^43, 46^**

	BW Digit Span (4)			**4 ^39, 42, 43, 46^**

	FW Dot Matrix (1)			**1 ^47^**

	BW Dot Matrix (1)			**1 ^42^**

	Faces task (1)	**1 ^41^**		

	Flanker (6)	**3 ^50, 52, 53^**		**3 ^42, 47, 55^**

	Frog Matrices (1)	**1 ^49^**		

	Go/No-Go task (1)			**1 ^45^**

	*N*-back task (2)		**1 ^39^**	**1 ^45^**

	Non-Word Repetition (2)		**1 ^46^**	**1 ^43^**

	Odd-One-Out (1)			**1 ^47^**

	Operation Span (1)	**1 ^56^**		

	Rotating Figure task (1)		**1 ^39^**	

	Simon (+ Simon-like; 5)	**2 ^56, 49^**	**1 ^55^**	**2 ^52, 54^**

	Simon Switching task (1)			**1 ^56^**

	Sky Search (2)	**2 ^47, 42^**		

IQ	RCPM (3)		**1 ^43^**	**2 ^39, 47^**


*Note*: Tasks are ordered by ability (EF, IQ), then alphabetically. ANT = Attention Network Task; BST = Bivalent Shape Task; DCCS = Dimensional Change Card Sort task; RCPM = Raven’s Colored Progressive Matrices.

#### Interim Discussion

The school-aged group was uniquely consistent in terms of the cognitive abilities measured, with all 23 experiments testing some component of EF. In addition to inhibition and attention, school-aged children were tested on WM much more frequently than infants or preschoolers (e.g., Cockcroft, 2016; Engel de Abreu, 2011). ***Table 2*** indicates that the most common EF tasks that were used among school-aged children were the ANT, Flanker task, and Simon tasks, which makes this group more comparable to adult bilinguals than the younger two age groups, in terms of tasks used. Bilingual advantages for performance on these EF tasks did not consistently emerge. More striking however, was that WM tasks rarely yielded any language group differences, with the exception of one experiment that reported a bilingual advantage for the Operation Span task (Tse & Altarriba, 2014), and two experiments that reported monolingual advantages for the Non-Word Repetition, *N*-back, and Rotating Figure tasks (Andreou et al., 2021; Engel de Abreu, 2011). WM tasks rarely yielded performance differences between bilinguals and controls in any age group. This corresponds to findings from previous meta-analyses which did not find evidence of a bilingual advantage for WM, in spite of advantages in other components of EF (Adesope et al., 2010; Gunnerud et al., 2020).

## Adolescent studies (13 to 18 years of age)

### Target cognitive abilities and cognitive tasks used

We identified three articles that tested adolescents. The cognitive abilities intended to be measured by these experiments fell into the following categories: executive function, general cognitive ability, and stimulus-in-noise perception. Executive function was assessed in two experiments (Kapa & Colombo, 2013; Gathercole et al., 2016). One of these assessed attentional control using the ANT (Kapa & Colombo, 2013). The other used the Simon task to examine interference suppression (Gathercole et al., 2016). Gathercole et al. (2016) also measured ‘general non-verbal cognitive ability’ using the Raven’s Progressive Matrices test. Stimulus-in-noise perception was measured by Krizman, Bradlow, Lam, & Kraus (2017), who tested adolescents on word-in-noise and tone-in-noise tasks. See ***Tables 5*** and ***6*** for further details.

#### Differences between bilinguals and controls

For a detailed breakdown of the measures and findings from each article in the adolescent age group see the Appendix. ***Table 4*** shows the differences found between bilingual and monolingual adolescent groups based on the task, with references to the Appendix number for each experiment.

**Table 4:** Adolescent Group Differences by Task Across Experiments.


COGNITIVE ABILITY	TASK (# OF EXPERIMENTS)	BILINGUAL ADVANTAGE EXPERIMENTS ^APPENDIX #^	MONOLINGUAL ADVANTAGE EXPERIMENTS ^APPENDIX #^	NO DIFFERENCES EXPERIMENTS ^APPENDIX #^

EF	ANT (1)	**1 ^59^**		

	Simon (1)			**1 ^58^**

Gen. Cog.	RPM (1)			**1 ^58^**

Stim.-in-Noise	Backward Masking (1)	**1 ^60^**		

	Simultaneous Masking (1)	**1 ^60^**		

	Word-In-Noise (1)			**1 ^60^**


*Note*: Tasks are ordered by ability (EF, general cognitive ability, stimulus-in-noise perception), then alphabetically. ANT = Attention Network Task; RPM = Raven’s Progressive Matrices.

#### Interim Discussion

The under-representation of adolescents in this literature was striking, with only three articles testing participants between 13 and 18 years of age compared to the large numbers of studies that tested younger children. We can speculate that this may be because adolescents are assumed to be more cognitively ‘adult-like’, given their close proximity in age to university students, who are the focus of much adult research. However, the cognitive abilities reviewed here are closely tied to the prefrontal cortex, the development of which is known to continue into an individual’s mid-twenties (Diamond, 2002). There is also evidence that key EF components such as working memory and shifting develop steadily into adolescence (Best & Miller, 2010). Because there were so few studies in this age group it was difficult to discern any particular pattern of abilities or tasks within this age group.

The experiments discussed in the preceding sections address a wide range of cognitive abilities using a large variety of tasks. For a summary of the abilities tested across the four age groups and the frequency with which each ability was measured, see ***Table 5***. As executive function was the most commonly tested ability by a significant margin, comprising approximately 82% of all experiments, ***Table 6*** was additionally created to present the tasks used to measure different components of EF across the four age groups.

**Table 5:** Percentage of Experiments for Each Category of Cognitive Ability in Across Age Groups.


AGE GROUP (# OF EXPERIMENTS)	COGNITIVE ABILITY – % (# OF EXPERIMENTS)							

	EXECUTIVE FUNCTION	MEMORY	THEORY OF MIND^b^	INTELLIGENCE^c^	CREATIVITY	GENERAL COGNITIVE ABILITY	STIMULUS-IN-NOISE PERCEPTION	INFORMATION PROCESSING

Infants (18)	**58.8%** (11)	**35.3%** (7)	**5.9%** (1)	–	–	**–**	–	**5.9**% (1)

Preschool (29^a^)	**86.2%** (25)	–	**24.1%** (7)	**3.4%** (1)	**3.4%** (1)	**3.4** (1)	–	–

School (23^a^)	**100.0%** (23)	–	–	**13.0%** (3)	–	**–**	–	–

Adolescents (3^a^)	**66.6%** (2)	–	–	–	–	**33.3%** (1)	**33.3%** (1)	**–**

% of Total Experiments (71^a^)	**83.1%** (59^a^)	**9.9%** (7)	**11.3%** (8)	**5.6%** (4)	**1.4%** (1)	**1.4%** (1^a^)	**1.4%** (1)	**1.4% (1)**


*Note*: As many experiments measured abilities from more than one category, the percentages for each ability in each age group always add up to more than 100%. ^a^ One experiment in the preschool group and one in the school group also tested adolescent participants (Gathercole et al., 2016; Kapa & Colombo, 2013). These experiments are represented in both the preschool/school and adolescent categories in this table, which is why the total number of experiments in the bottom row is fewer for EF and General Cognitive Ability than the sum of those experiments across all age groups. ^b^ The Theory of Mind category includes perspective-taking tasks. ^c^ The Intelligence category includes abstract reasoning.

**Table 6:** Measures Used Across Components of Executive Function by Age Group for Infants, Preschool, and School Children.


MEASURES	EF COMPONENT																	

	INFANTS				PRESCHOOL							SCHOOL						

	ATTN.^a^	INHIB.^b^	CF	WM^d^	ATTN.^a^	INHIB.^b^	CF	SHIFT.^c^	MON.	‘COGNITIVE CONTROL’	WM^d^	ATTN.^a^	INHIB.^b^	SHIFT.^c^	MON.	‘COGNITIVE CONTROL’	WM^d^

**ANT**					17, 35, 37, 38	20						40, 46, 54, 57, 59			57		

**Bivalent Shape**													45				

**Corsi Blocks**																	50

**Counting Recall**																	43,46

**C-TONI**						20											

**DCCS**					36	15, 18, 19, 20, 36	23, 24, 26	15, 25, 32, 36	36				44, 51	44, 50	44		

**Delay**		8, 12				17,20,36											

**Delay of Gratification**						20											

**FW Digit Span**											25, 32						43, 46

**BW Digit Span**											25, 32, 34						39, 42, 43, 46

**FW Dot Matrix**																	47

**BW Dot Matrix**																	42

**ECBQ**	14	14															

**Faces**													41	41			

**Flanker**													42, 47, 50, 52, 53			55	

**Frog Matrices**													49				

**Go/No-Go**						17							45				

**Hide the Pots**				4													

**K-CPT-2**					21												

**KRISP**						20											

**Listening Span**											31, 33						

**Luria Stage 1**										16							

**Luria Stage 2**										16							

**Moving Word**					18												

**Multilocation**		8, 12		8													

**N-back**																	39,45

**NSND**						28											

**Non-Word Repetition**																	43,46

**Odd-One-Out**																	47

**Operation Span**																	56

**Pattern Recall**											33						

**Reverse Cat.**		8, 12	8														

**Rotating Figure**																	39

**Search tasks**	14											42, 47					

**Simon Says**						20, 23, 24, 36											

**Simon (+ ‘Simon-like’)**					33	32, 58						54, 56	49, 52			55	49

**Simon Switching**														45			

**Spatial Conflict**		14															

**Spin the Pots**				5													

**Statue**						20											

**Stroop (+ ‘Stroop-like’)**		8, 12				23, 24, 25, 34, 36							49				

**TEC**					26												

**VExCP**	7,9	10															

**Visually Cued Recall**						20											


*Note*: Numbers indicate the Appendix entry for each article. Adolescent experiments are omitted from this table due to the very small number of experiments that tested EF in this population. The two experiments that did measure EF in adolescents measured attention using the ANT (Kapa & Colombo, 2013) and inhibition using the Simon task (Gathercole et al., 2016). ANT = Attention Network Task; CF = Cognitive flexibility; C-TONI = Comprehensive Test of Nonverbal Intelligence; DCCS = Dimensional Change Card Sort Task; ECBQ = Early Childhood Behavior Questionnaire; K-CPT-2 = Conner’s Kiddie Continuous Performance test – Second Edition; KRISP = Kansas Reflection/Impulsivity Scale; NSND = Non-Symbolic Numerical Discrimination task; TEC = Tasks of Executive Control; VExCP = Visual Expectation Cueing Paradigm (also referred to as an ‘anticipatory looking paradigm’); WM = Working memory. ^a^ Experiments assessed different types of attention, including ‘controlled attention’; ‘selective attention’; ‘attentional flexibility’; and ‘attention allocation’. ^b^ Experiments assessed different types of inhibition, with some forms of inhibition being referred to using different terms in different articles. The types of inhibition assessed included ‘inhibitory control’ (also ‘interference suppression’); ‘response suppression’ (also ‘response inhibition’); and ‘response control’. ^c^ Shifting was also referred to as ‘task switching’ or ‘switching’ in some experiments. ^d^ Working memory was also referred to as ‘updating’ in some experiments.

### Comment on excluded papers

217 articles were excluded at the full-text stage of our review. An Excel document listing all the excluded papers with the reasons for exclusion can be found at *https://doi.org/10.5683/SP2/CBU7LL*. Of these, 106 articles either did not report information about the socioeconomic status (SES) of the participants (or did not control for unmatched SES between groups), or did not provide sufficient information about the language background of the bilingual participants. Eighty-three articles, over a third of the articles excluded at this stage, were deemed ineligible exclusively for one or both of these reasons. The majority of articles excluded for giving insufficient language background information did not provide information about the age at which bilingual participants acquired their L2, and several did not report the languages spoken by a group of heterogeneous bilinguals. The inconsistency with which information about SES and bilingual language characteristics is reported in studies on bilingualism and cognition has been noted by previous authors (Adesope et al., 2010; Gunnerud et al., 2020; Ladas et al., 2015). SES is known to affect cognitive performance, with higher SES being linked to cognitive benefits (Noble, Norman, & Farah, 2005). It is also important to provide detailed language background information for bilingual participants, as the cognitive effects of bilingualism could differ depending on the extent to which a speaker uses his or her second language, the proficiency in each language, and the age at which the second language was acquired (Kapa & Colombo, 2013; Luk et al., 2011).

## General Discussion

The main goal of the present review was to examine what research most commonly refers to when measuring non-linguistic ‘cognition’ in simultaneous bilingual children. The most frequent age group studied in the reviewed articles was preschool-aged children (2;6–6 years), followed by school-aged children (6–12 years), infants (0–2;6 years), and adolescents (13–18 years). Across all the age groups examined, by far, the most frequently assessed cognitive ability was executive function (EF), a trend that parallels the adult bilingual literature. Beyond EF, quite a few experiments focused on memory and theory of mind (ToM). In addition, a small number of studies assessed intelligence, creativity, stimulus-in-noise-perception, and information processing as different facets of cognition. This is the pattern across studies that measured cognition in simultaneous bilingual children and reported sufficient detail about language background and socioeconomic status. As highlighted in the Results, more than 80 articles were ineligible for the current review only because they did not share sufficient background details about their participants, therefore we are unable to report what aspects of cognition those studies considered.

### Target cognitive abilities

For the infant, preschool, and school-aged groups, the most frequently targeted EF components were *inhibition* and *attention*. The two experiments that measured EF in adolescents also focused on inhibition (Gathercole et al., 2016) and attention (Kapa & Colombo, 2013) respectively. The majority of articles that targeted inhibition measured *interference suppression*, the ability to suppress distracting or conflicting information in order to give a correct response (e.g., Kovacs & Mehler, 2009; Mehrani & Zabihi, 2017; Pino Escobar et al., 2018). It has been consistently shown that bilinguals and monolinguals perform differently on this aspect of inhibition, but generally demonstrate no significant differences for response inhibition, the ability to withhold a pre-potent response (Barac et al., 2016; Carlson & Meltzoff, 2008). Indeed, this was the overall pattern of results that emerged across the studies we reviewed. For attention, studies most frequently measured the three attention functions assessed by the Attention Network Task, that is, alerting, orienting, and control of attention (e.g., Barac et al., 2016; Ladas et al., 2015), or selective attention (e.g., Bosma et al., 2017; Verhagen et al., 2020). The focus on the attention network in children again mirrors the literature on adults, where these functions are the target of a large number of studies (Lehtonen et al., 2018).

The examination of some EF components (e.g., inhibition, attention control) based on behavioural responses is highly feasible in older children given the developmental trajectory of the various EF components (Diamond, 2013) and the well-established tests that are available for this age group. However, as discussed previously, it is not always clear whether these abilities can be observed in infants younger than one year. At this age, attentional orienting is primarily related to distress regulation (e.g., Harman, Rothbart, & Posner, 1997); it can also reflect habituation or preference (Hunter & Ames, 1988); infants’ attention can be also conditioned at this age (Werker, Polka, & Pegg, 1997). However, signs of more (self-) controlled attention (i.e., inhibition, cognitive control) only seem to appear at the end of the first year (e.g., Diamond, 2013). Only a very small number of studies have demonstrated rudimentary forms of EF before 12 months of age (e.g., 6–7 months of age: Sheese, Rothbart, Posner, White, & Fraundorf, 2008; 10 months: Blankenship, Slough, Calkins, Deater-Deckard, Kim-Spoon, & Bell, 2019). While the presence of certain EF components (i.e., inhibition, attention control) during the first year of life is still debatable and there is a lack of established behavioural measures of these components during this period, six experiments assessed whether bilingual exposure affects such abilities in infants under 12 months (Comishen et al., 2019, Studies 1 & 2; Kalashnikova et al., 2020; Kovacs & Mehler, 2009, Experiments 1–3). The presence of a bilingual cognitive advantage before one year of age in some of these studies was interpreted as bilingualism accelerating the onset of EF. It has been also suggested that growing up in a bilingual environment may change the way attention is allocated in early infancy due to the presence of two contrasting communication systems (see Bialystok, 2015), which could be a precursor to EF in early childhood.

While inhibition and attention control have been highlighted in the bilingual advantage literature as being enhanced by bilingualism, this is generally believed to result from the demands of inhibiting one language while actively attending to and using the other, as well as switching languages as context requires (e.g., Bialystok et al., 2012). It is not clear why these same processes would be expected to benefit from bilingualism in infants who have presumably not yet reached a stage where they are actively controlling their languages. Especially in the case of infants younger than 12 months, to the best of our knowledge, there is currently no research suggesting that infants are capable of functionally distinguishing their languages. Bilingual newborns and 4-month-olds are able to perceptually distinguish between the languages in their environment, just like their monolingual peers can distinguish between different languages (Bosch & Sebastian-Galles, 2001; Byers-Heinlein et al., 2010; Molnar et al., 2014). However, perceptual discrimination does not equate to the functional discrimination that is a prerequisite to switching. As an example, some unpublished research has found that bilingual infants younger than 12 months in one-parent, one-language households do not reliably associate each language with the parent who speaks it (Molnar & Carreiras, 2014, 2015). Hence, currently it is still unclear if preverbal infants can actively switch between their languages or inhibit a language if needed.

Compared to inhibitions and attention, working memory was only tested in two experiments with infants. Although the progression of WM development is slower than simple short-term memory, young infants are able to update the contents of WM in order to correctly locate a toy when its location changes in a simple WM measure like the A-not-B task (Diamond, 2013), yet this task was not used in any of the infant experiments we reviewed. This arguably represents a missed opportunity to assess bilingual/monolingual differences on a task that is better established to measure cognition in this age group. Across all studies we reviewed, the frequency of experiments targeting WM rose noticeably as participants aged, a trend that corresponds to the established finding that WM shows a slower developmental progression in childhood than short-term or procedural memory, with school-aged children having a greater ability to perform complex WM tasks than younger children or infants (Cowan, AuBuchon, Gilchrist, Ricker, & Saults, 2011; Diamond, 2013; Luciana & Nelson, 1998).

### Most common cognitive tasks used

We also reviewed the specific tasks that are used to measure the different cognitive abilities. EF was measured with a huge variety of tasks compared to other cognitive abilities, with a total of 30 different tasks or task types (e.g., ‘Simon-like’ and ‘Stroop-like’ tasks) across the four age groups. As noted previously, tasks became increasingly comparable to those used in the adult literature as participants entered the school age range, in line with proposed developmental trajectories for EF in which dramatic changes occur in the first 5 years, followed by gradual improvements in the complexity of tasks that can be performed and the accuracy and speed of performance (Best & Miller, 2010). EF tasks common in the school group included the Flanker task, Simon task, and Attention Network Task (the ANT was also relatively common in the preschool group). In comparison, the most frequent EF tasks in younger groups included the Reverse Categorization and Visual Expectation Cueing Paradigm in infants, and the Dimensional Change Card Sort and Simon Says tasks in preschool children.

The Flanker and Simon tasks are ubiquitous throughout the literature on bilingualism. However, the assumption that these tasks are measuring the same facet of cognition (generally inhibition) has been questioned, with some finding evidence that children’s performance on the Flanker and Simon tasks does not correlate (Poarch, 2018; Poarch & van Hell, 2019). Similar issues of convergent validity among these common EF tasks have been raised in the adult bilingual literature (Paap & Greenberg, 2013). Given that these tasks are used throughout the child and adult literature to support or contradict the notion of a bilingual advantage in EF specifically, clarifying whether they are in fact tapping the same or similar processes is of key importance.

Working memory was measured using Forward Digit Span and Non-Word Repetition tasks in several of the reviewed publications (Bialystok & Martin, 2004, Study 1 & 2; Cockcroft, 2016; Engel de Abreu, 2011; Mehrani & Zabihi, 2017). However, it is not clear that these tasks actually do measure WM, as they only require maintaining a list of items in memory and recalling them verbatim rather than manipulating the information (Diamond, 2013). The backward version of this task imposes more of a demand on WM, and was also used in some experiments we reviewed. There was a lack of agreement among publications about what the Forward Digit Span and Non-Word Repetition tasks measure – in addition to the experiments that used them to target WM, two experiments identified the Forward Digit Span task as a measure of short-term memory (Diaz & Farrar, 2018a; Diaz & Farrar, 2018b) and another experiment used both tasks as short-term memory rather than WM measures (Namazi & Thordardottir, 2010).

The tasks that were used most frequently across all age groups were the Dimensional Change Card Sort task, Attention Network Task, Simon and Simon-like tasks, and Stroop and Stroop-like tasks. Stroop and Stroop-like (e.g., Day/Night, Happy/Sad) tasks were consistently identified as measures of inhibition, though the specific subtype of inhibition varied. Diaz & Farrar (2018b) identified the Day/Night Stroop as measuring ‘inhibitory control’, another term for interference suppression. On the other hand, Tran and colleagues (2019) described this task as measuring response inhibition, though one could argue that this categorization misrepresents the Day/Night task, since the task requires withholding a pre-potent response and giving a conflicting one, i.e., interference suppression.

Like Stroop tasks, the ANT was also highly consistent in terms of the identified target ability, with all but one experiment targeting attention or attentional control. One experiment that used the ANT identified inhibition as the cognitive ability of interest (Carlson & Meltzoff, 2008). The DCCS and Simon-style tasks were two of several tasks for which researchers described different cognitive target abilities depending on the experiment, or for which multiple target abilities were identified in a single experiment. This trend was particularly prominent for the DCCS, which was identified as a measure of attention, inhibition, cognitive flexibility, shifting, or monitoring depending on the experiment. The Simon task, and a Simon-type task called the ‘Pictures task’ (Morales et al., 2013), was described as measuring attention, inhibition, and ‘cognitive control’ by different researchers.

Other tasks whose cognitive targets were identified differently across studies included the Visual Expectation Cueing Paradigm (attention and inhibition), the Reverse Categorization task (inhibition and cognitive flexibility), the Multilocation task (inhibition and WM), and the Faces task (inhibition and ‘switching’). Such variation in targeted abilities for the same tasks may be inevitable due to the fact that the different EFs are highly interrelated, rarely, if ever, operating in isolation (Best & Miller, 2010; Diamond, 2013). Thus, constructing a task that is a “pure” measure of only one EF component is very difficult. Tasks that are usually identified as measuring inhibition (e.g., Simon task), for example, also involve selective and sustained attention to information relevant for success, and a task that measures shifting (like the DCCS) involves inhibition of the previous set of rules once the switch occurs. With this in mind, it is a reasonable trend in the present review that there was not a one-to-one correspondence between cognitive ability and task for EF in particular.

### Differences between bilinguals and monolinguals

We also compared the frequency of bilingual vs. monolingual cognitive differences observed within tasks and age groups. Generally speaking, results seem highly variable and a consistent bilingual advantage pattern across the studies did not emerge (see Gunnerud et al. (2020) for a recent meta-analysis on this topic). This is in spite of the fact that the studies we reviewed matched participants on SES and age of acquisition, and recruited bilinguals with approximately equal proficiency in both languages. This pattern of mixed results is consistent with findings from previous reviews and meta-analyses (Gunnerud et al., 2020; Lehtonen et al., 2018; Paap et al., 2015; though also see Grundy, 2020). Current debates focus on the question of the circumstances in which a bilingual cognitive advantage develops.

One consistent difference that did emerge in EF components across age groups was that bilinguals tended to outperform monolinguals on tasks measuring interference suppression, but did not tend to perform differently from monolinguals on tasks measuring response inhibition (e.g., Barac et al., 2016; Carlson & Meltzoff, 2008). This is a similar pattern to what emerged in the meta-analysis by Gunnerud et al. (2020), in which “hot inhibition” tasks that can be considered to measure response inhibition (e.g., Gift Delay) did not show any bilingual advantage, whereas “cold inhibition”, including interference suppression tasks such as Stroop and Simon, did show some evidence of an advantage (albeit a weak one). This pattern may occur because interference suppression tasks more closely imitate the kind of control bilinguals must exert when speaking one of their languages, that is, inhibiting the language that is not relevant in a given context in order to correctly select the target language (Bialystok & Martin, 2004; Bialystok et al., 2012). Constant practice selectively attending to one language over the other may lead to improved performance on tasks that require inhibiting distracting information in order to correctly respond.

In the infant group, EF tasks overall were reported to yield no differences between language groups much more often than they yielded a bilingual advantage. This pattern may be in line with our previous discussion point that looking for bilingual EF advantages in such young children might be problematic, given the lack of clarity about the extent to which certain EF components are present in this age group and the lack of well validated behavioural tasks for measuring it (Diamond, 2013). In this particular age group, measuring the neural mechanisms underlying the development of EF might be an option, to avoid the limitations associated with overt responses required by behavioural tasks.

In contrast to EF tasks, the Deferred Imitation task used to assess memory flexibility (MF) in infants was reported to yield an advantage for bilinguals in every experiment that used it. The lack of a clear EF advantage paired with an advantage for MF in bilingual infants could raise questions about why the latter advantage may occur. In their first study of MF in bilingual and monolingual children, Brito and Barr (2012) suggest that enhanced EF in bilingual infants may lead to generalized benefits for other cognitive abilities like MF, but if current evidence does not support clearly that EF is enhanced, the reason for a bilingual advantage for MF in infancy would require further examination. This point, as well, raises the question of whether our current methods assessing infants’ EF are adequate.

### Terminology used across studies

Throughout the reviewed literature, it was common that different articles used different terms to refer to the same cognitive ability. This was particularly true for research on executive function, which may be a consequence of the challenge of defining EF and the multiple conceptualizations that exist (Miyake et al., 2000; Miyake & Friedman, 2012; Jurado & Rosselli, 2007). There were several examples of variable terminology in the EF experiments we reviewed. Executive function itself was also referred to as ‘executive control’, ‘cognitive control’, and ‘attentional control’, depending on the study. The ability to suppress conflicting information in order to give a correct response was alternately called ‘interference suppression’, ‘interference inhibition’, ‘conflict inhibition’ and ‘inhibitory control’. ‘Response inhibition’ and ‘response suppression’ were both used to refer to the ability to withhold a pre-potent response. The terms ‘shifting’, ‘task shifting’, ‘task switching’, and ‘cognitive flexibility’ were all used to refer to the ability to switch between task dimensions or rules. Though the variety of terms for different abilities do not necessarily impede comprehension of individual papers, it is sometimes unclear when comparing studies whether these terms are fully interchangeable or if they represent functions at different levels of an EF hierarchy. For example, in some studies we reviewed, “attentional control” seemed to refer to an attention-specific component under the umbrella of more general “executive functioning” or “cognitive control” (e.g., Kalashnikova et al., 2020; Kapa & Colombo, 2013; Ladas et al., 2015; Poarch & van Hell, 2012), whereas in others the term seemed to encompass a broader range of executive functions (e.g., Tse & Altarriba, 2014).

## Conclusions

Here, we surveyed the non-linguistic cognitive measures used with simultaneous bilingual populations from infancy to adolescence. Our review took a broader look at the literature than Takakuwa (2000) when examining the meaning of ‘cognition’ in developing bilinguals, and identified a diverse range of cognitive abilities that are tested in this population. The results indicate that the developmental literature is much like the literature on adult bilinguals, with executive function emerging as the most commonly targeted aspect of cognition across all age groups. This is true even when the infant age group is considered, despite that only a few studies demonstrated some rudimentary forms of EF during the first year of life – irrespective of the question of the bilingual cognitive advantage. In contrast to EF, investigations of memory flexibility and theory of mind in infants and preschool-aged children align with established developmental trajectories. Most tasks were consistent in terms of targeted abilities, with the exception of some EF tasks that vary in terms of what studies use them to measure, reflecting the difficulty of isolating the components of EF from one another. Further, surprisingly, very few studies focused on adolescents, despite the fact that cognitive abilities are not necessarily ‘adult-like’ at this age yet. In sum, due to the ongoing debate regarding the bilingual cognitive advantage, studies assessing cognition in developmental monolingual and bilingual populations are increasing. This expansion in cognition research highlights current issues in defining certain aspects of cognition (e.g., executive function) and the need for more basic research to understand the developmental trajectory of some cognitive functions from infancy to adolescence.

## Future Directions

Based on the findings of this systematic review, the following recommendations for future empirical research with bilingual children emerged:

- Given the number of tasks whose cognitive targets were identified differently across studies (e.g., the DCCS), as well as the tasks that may suffer from inconsistent convergent validity (e.g., Simon and Flanker tasks), more research may be needed to clarify the specific cognitive abilities that common tasks are measuring across the life-span and to determine whether tasks that are frequently used to measure the same processes actually do so.
- Partially related to our first point, we recommend more standardization of terminology used for the components of executive function in future research. Currently several different terms are used for EF as a whole and for its component abilities. More consistency in terminology would facilitate the comparison of results across studies, allowing for a more complete understanding of the operation of executive processes across different experimental populations.
- Based on the emerging theories of bilingual cognitive advantage, we also argue that future research should look beyond EF when measuring cognition in developing bilinguals, given the preponderance of research that has focused on EF to date. In infants, this could mean more research aimed at elucidating the neural precursors to EF, investigating the role of attention allocation in bilingual infants, or at explaining why bilingual infants may exhibit greater memory flexibility than monolinguals.
- In older children, studies could examine bilinguals’ theory of mind or creativity in greater depth. In the context of the bilingual advantage literature, some have pointed out that statistical differences in EF abilities may have limited relevance in everyday life (see Poarch & Krott, 2019). On the other hand, advantages for ToM and creativity, which have received less attention (with the exception of ToM in preschool children), may have more noticeable effects in bilinguals’ daily lives.
- Finally, we think that cognition in adolescent bilinguals merits greater attention, as cognitive abilities are still developing between ages 13 and 18.

## Additional File

The additional file for this article can be found as follows:

10.5334/joc.184.s1 Appendix. All reviewed articles, arranged by age group.

## Footnotes

A more in-depth quantitative treatment of this topic is available in Gunnerud and colleagues’ (2020) recent meta-analysis.

We thank an anonymous reviewer who helped us to identify one eligible article that did not appear in any database or manual searches for this review.
