# Supplementary material for: Measures of Bilingual Cognition – From Infancy to Adolescence
Source: J Cogn. 2021 Aug 26;4(1):45. doi: 10.5334/joc.184 (PMC8396129; doi:10.5334/joc.184)
Supplement: Appendix. — All reviewed articles, arranged by age group. [file joc-4-1-184-s1.pdf]

## Appendix

*All reviewed articles, arranged by age group. Publications testing homogenous bilingual participants are indicated with 'HM' in the first column, and those testing heterogeneous bilingual groups are indicated with 'HT'. Any interpretive comments on the results of experiments come from the articles themselves.*

| # | Article information | Participant Age | Controls | Bilingual Characteristics | Aspects of Cognition | Primary Outcome Measures | Results |
|---|---|---|---|---|---|---|---|
| **INFANTS** | | | | | | | |
| **1**<br><br>**HT** | Barr, Rusnak, Brito, & Nugent. (2020). Actions speak louder than words: Differences in memory flexibility between monolingual and bilingual 18-month-olds. | 18 months | Monolinguals (English)<br><br>+<br><br>Baseline control group of monolinguals and bilinguals (did not see the demonstration of target actions) | Languages:<br>English<br>+<br>Spanish<br>Chinese<br>Portuguese<br>German<br>Korean<br>Polish<br>(+ 4 additional languages)<br><br>Language exposure:<br>Exposed to L2 a minimum of 20% of the time when awake | Memory flexibility (MF)<br><br>Cued recall (CR) | MF + CR:<br>Deferred imitation memory generalization task<br><br>2 conditions:<br>- Label (to provide novel discriminative label for each stimulus)<br>- No label | Monolingual and bilingual groups in both conditions outperformed baseline control group for cued recall (i.e. were able to recall target actions when stimulus objects were the same as the demonstration).<br><br>Only bilingual groups (in both conditions) outperformed baseline controls on memory generalization (i.e. performed target actions on different stimulus objects).<br><br>No differences between bilinguals and monolinguals on cued recall.<br><br>Significant difference in favour of bilinguals for generalization. |
| **2**<br><br>**HT** | Brito & Barr. (2012). Influence of bilingualism on memory generalization during infancy. | 18 months | Monolinguals (English, Spanish, Portuguese)<br><br>+<br><br>Monolingual baseline controls (did not see the demonstration of target actions) | Languages:<br>One of:<br>English<br>Spanish<br>Hebrew<br>+<br>Spanish<br>Portuguese<br>Hebrew<br>German<br>Cantonese<br><br>Language exposure:<br>Mean L1 = 63%<br>Range L2 = 25-50% | Memory flexibility (MF) | Deferred imitation memory generalization task | Bilingual infants had significantly higher imitation scores at test than monolingual infants: 9/15 bilingual infants imitated the previously demonstrated actions with the new puppet, compared to only 1/15 monolinguals. |

| | | | | | | | |
|---|---|---|---|---|---|---|---|
| **3**<br><br>**HT** | Brito & Barr. (2014). Flexible memory retrieval in bilingual 6-month-old infants. | 6 months | Monolinguals (English)<br><br>+<br><br>Monolingual baseline control group (did not see the demonstration of target actions) | Languages:<br>One of:<br>English<br>German<br>Spanish<br>Russian<br>Portuguese<br>Greek<br>(+ 2 additional languages)<br>+<br>English<br>Spanish<br>Arabic<br>Hebrew<br>French<br>Mandarin<br>Hungarian<br><br>Language exposure:<br>Mean L1 = 69%<br>Range L2 = 20-50% | Memory flexibility (MF) | Deferred imitation memory generalization task<br><br>2 puppet conditions:<br>- 1 feature change from demonstration to test<br>- 2 feature change | 1 feature change condition: Both monolinguals and bilinguals were able to generalize actions from demonstration to test.<br><br>2 feature change condition: Only bilinguals were able to generalize. |
| **4**<br><br>**HT** | Brito, Greaves, Leon-Santos, Fifer, & Noble. (2020). Associations between bilingualism and memory generalization during infancy: Does socioeconomic status matter? | 18 months | Monolinguals (English, Spanish, Bulgarian, Korean)<br><br>+<br><br>Monolingual baseline control group (did not see the demonstration of target actions) | Languages:<br>One of:<br>Spanish<br>English<br>German<br>Hungarian<br>French<br>Tagalog<br>Vietnamese<br>+<br>English<br>Spanish<br>Catalan<br>Amharic<br>Cantonese<br>Mandarin<br>Murathi<br><br>Language exposure:<br>Mean L1: 63%<br>Range L2: 25-50% | Memory flexibility (MF)<br><br>Cued recall (CR)<br><br>Working memory (WM) | MF + CR:<br>Deferred imitation memory generalization task<br><br>WM:<br>Hide the Pots | * Recruited low- and high-SES groups of monolinguals and bilinguals in order to assess impact of SES on MF.<br><br>CR:<br>No significant effect of bilingualism emerged.<br><br>MF:<br>Both low-SES and high-SES bilinguals significantly outperformed low- and high-SES monolingual groups and the baseline control group.<br><br>WM:<br>No significant effect of bilingualism emerged. |

| | | | | | | | |
|---|---|---|---|---|---|---|---|
| **5**<br><br><br><br>**HT** | Brito, Grenell, & Barr. (2014). Specificity of the bilingual advantage for memory: Examining cued recall, generalization, and working memory in monolingual, bilingual, and trilingual toddlers. | Mean = 24.5 months | Monolinguals (English)<br><br>+<br><br>Monolingual baseline controls (did not see the demonstration of target actions) | <u>Languages</u>:<br>12 different languages represented in different combos of 2 or 3 depending on language group<br><br><u>Language exposure:</u><br>Bilinguals:<br>Mean L1 = 69%<br>Range L2 = 25-50%<br><br>Trilinguals:<br>Mean L1 = 48%<br>Range L2 = 25-40%<br>Range L3 = 10-30% | Cued recall (CR)<br><br>Memory flexibility (MF)<br><br>Working memory (WM) | <u>CR & MF:</u><br>Deferred imitation memory generalization task<br><br><u>WM:</u><br>Spin the Pots task | <u>CR</u>: No significant differences between language groups, though all performed better than baseline group.<br><br><u>MF</u>: Only bilingual group significantly outperformed baseline group.<br><br><u>WM</u>: No significant differences between language groups. |
| **6**<br><br><br><br>**HM**<br>**+**<br>**HT** | Brito, Sebastian-Galles, & Barr. (2015). Differences in language exposure and its effects on memory flexibility in monolingual, bilingual, and trilingual infants. | **<u>Experiments 1 & 2:</u>**<br><br>18 months | **<u>Experiment 1</u>**<br><br><u>Washington</u>:<br>Monolinguals (English)<br>+<br><br>Monolingual baseline controls (did not see the demonstration of target actions)<br><br><u>Barcelona:</u><br>Monolinguals (Spanish or Catalan)<br><br><br>**<u>Experiment 2</u>**<br><br>Groups from Experiment 1 | **<u>Experiment 1</u>**<br><br><u>W: Languages:</u><br>English<br>+<br>Spanish<br><br><u>W: Language exposure:</u><br>Mean L1 = 68%<br>Range L2 = 20-50%<br><br><u>B: Languages:</u><br>One of:<br>Spanish<br>Catalan<br>+<br>Spanish<br>Catalan<br><br><u>B: Language exposure:</u><br>Mean L1 = 68%<br>Range L2 = 25-45%<br><br>**<u>Experiment 2</u>**<br><br><u>W&B: Languages:</u><br>14 different languages represented in different combos of 3<br><br><u>Language exposure:</u><br>Trilinguals:<br>Mean L1 = 53% | **<u>Experiments 1 & 2:</u>**<br><br>Memory flexibility (MF) | **<u>Experiments 1 & 2:</u>**<br><br><u>MF:</u><br>Deferred imitation memory generalization task | **<u>Experiment 1</u>**<br><br><u>MF</u>: Both groups of bilinguals demonstrated better MF than monolingual groups:<br>- Bilinguals correctly imitated more actions at test than monolinguals<br>- No effect of language pairing (e.g. English-Spanish vs. Spanish-Catalan) for bilingual performance<br><br>**<u>Experiment 2</u>**<br><br><u>MF</u>: Trilingual participants did not show any advantage for imitating actions with unfamiliar puppet compared to baseline control group (thus, only bilinguals showed a MF advantage in this study). |

| | | | | | | | |
|---|---|---|---|---|---|---|---|
| | | | | Mean L2 = 30%<br>Mean L3 = 18% | | | |
| **7**<br><br>**HT** | Comishen, Bialystok, & Adler. (2019). The impact of bilingual environments on selective attention in infancy. | **Study 1 & 2:**<br><br>6 months | **Study 1:**<br><br>Monolinguals (English)<br><br>**Study 2:**<br><br>Monolinguals (English) | **Study 1:**<br><br>Languages:<br>English<br>+<br>Cantonese<br>Czech<br>Gurati<br>Italian<br>Korean<br>Persian<br>(+ 4 additional languages)<br><br>Language exposure:<br>Mean L2 = 60.8% daily<br><br>**Study 2:**<br><br>Languages:<br>English<br>+<br>Arabic<br>Cantonese<br>French<br>Italian<br>Mandarin<br>Portuguese<br>(+ 5 additional languages)<br><br>Language exposure:<br>Mean L2 = 45.8% daily | **Study 1:**<br><br>- Selective attention: "Ability to form visual expectations"<br><br>**Study 2:**<br><br>- Selective attention<br>- Attentional flexibility | **Study 1:**<br><br>Visual Expectation Cueing Paradigm (VExCP)<br><br>**Study 2:**<br><br>Same as Study 1 but blocks differed:<br>Block 1: Trials had predictable cues<br>Block 2: Cues predicted the opposite location from Block 1 | **Study 1:**<br><br>No significant differences were found in bilingual and monolingual infants' ability to form visual expectations: no significant difference in percentage of correct anticipatory eye movements following predictable cue.<br><br>**Study 2:**<br><br>No significant difference in rate of correct anticipations for bilinguals or monolinguals. However, bilinguals performed above chance post-switch, whereas monolinguals did not.<br><br>Bilinguals had faster reactive latencies post-switch than monolinguals, suggesting that bilinguals were more efficient at updating expectations. |

| | | | | | | | |
|---|---|---|---|---|---|---|---|
| **8**<br><br>**HM** | Crivello, Kuzyk, Rodrigues, Friend, Zesiger, & Poulin-Dubois. (2016). The effects of bilingual growth on toddlers' executive function. | 2 waves to examine effect on EF of having more translation equivalents (TEs)<br><br>Wave 1: 24 months<br><br>Wave 2: 31 months | Monolinguals (English) | Languages:<br>One of:<br>English<br>French<br>+<br>French<br>English<br><br>Language exposure:<br>Wave 1: Mean L2 = 35.5%<br><br>Wave 2: Mean L2 = 36% | Executive function:<br>- Cognitive flexibility (CF)<br>- Inhibitory control (IC)<br>- Response suppression (RS)<br>- Working memory (WM) & response control (RC)<br><br>(Note: assessed EF at Wave 2 only; only TEs assessed at Wave 1) | "Conflict tasks":<br>- Reverse Categorization (CF, IC)<br><br>- Shape Stroop (IC)<br><br>Other tasks:<br>- Gift delay task (RS)<br><br>- Multilocation task (WM/RC) | Conflict tasks: Bilinguals showed *marginally significant* ($p$ = 0.05) superior performance compared to monolinguals.<br><br>Gift Delay: No significant difference between bilinguals and monolinguals.<br><br>Multilocation: No significant differences between bilinguals and monolinguals.<br><br>Influence of TEs for bilinguals: A larger increase in # of TEs significantly predicted better performance on conflict EF tasks, but not other two EF tasks. |
| **9**<br><br>**HM** | Kalashnikova, Pejovic, & Carreiras. (2020). The effects of bilingualism on attentional processes in the first year of life. | 7 months | Monolinguals (Spanish or Basque) | Languages:<br>Spanish<br>+<br>Basque<br><br>Language exposure:<br>Dominant language range = 50.4-74.7%<br><br>Non-dominant language range = 25.3-49.6%<br><br>Exposure to both languages at home and in the community | Executive function:<br>- Attentional control | Anticipatory looking paradigm* with auditory and visual cue conditions<br><br>*Identical to Visual Expectation Cueing Paradigm used in Kovacs & Mehler, 2009 | Auditory condition:<br>Bilinguals fixated the reward itself significantly more in the post-switch phase than monolinguals.<br><br>Monolinguals were more likely to fixate the correct location in the post-switch phase than bilinguals, but this was found to reflect chance performance by monolinguals and below-chance performance from bilinguals rather than indicating a monolingual advantage/bilingual disadvantage per se.<br><br>Visual condition:<br>No significant effects emerged.<br><br>Extent of exposure to non-dominant language did not impact performance in experimental paradigm. |

| | | | | | | | |
|---|---|---|---|---|---|---|---|
| **10**<br><br>**HT** | Kovacs & Mehler. (2009). Cognitive gains in 7-month-old bilingual infants. | **<u>Experiments 1, 2, & 3:</u>**<br><br>7 months | **<u>Experiment 1</u>**<br><br>Monolinguals (Italian)<br><br>**<u>Experiment 2</u>**<br><br>Same characteristics as Exp. 1<br><br>**<u>Experiment 3</u>**<br><br>Same characteristics as Exp. 1 & 2 | **<u>Experiment 1</u>**<br><br><u>Languages:</u><br>Italian<br>+<br>Slovenian<br>Spanish<br>English<br>Arabic<br>Danish<br><br><u>Language exposure:</u><br>Parents had different mother tongues<br><br>Exposed to both languages daily<br><br>**<u>Experiment 2</u>**<br><br><u>Languages:</u><br>Italian<br>+<br>Slovenian<br>Spanish<br>English<br>French<br><br><u>Language exposure:</u><br>Same characteristics as Exp. 1<br><br>**<u>Experiment 3</u>**<br><br><u>Languages:</u><br>Italian<br>+<br>Slovenian<br>Spanish<br>English<br>French<br>Russian<br><br><u>Language exposure:</u><br>Same characteristics as Exp. 1 & 2 | **<u>Experiment 1</u>**<br><br>Executive function:<br>- Inhibitory control<br><br>**<u>Experiment 2</u>**<br><br>Same as Exp. 1<br><br>**<u>Experiment 3</u>**<br><br>Same as Exp. 1 & 2 but with visual instead of auditory cues | **<u>Experiment 1</u>**<br><br>Visual Expectation Cueing Paradigm (VExCP)<br><br>**<u>Experiment 2</u>**<br><br>VExCP but different cue structure in pre- and post-switch phases<br><br>**<u>Experiment 3</u>**<br><br>VExCP with visual cues instead of speech cues | **<u>Experiment 1</u>**<br><br>Bilinguals increased correct anticipatory looks to new location and decreased perseverative responses to old location post-switch.<br><br>No significant difference between pre- and post-switch for monolinguals.<br><br>**<u>Experiment 2</u>**<br><br>Same as Exp. 1: Bilinguals increased correct anticipations and decreased perseverative responses post-switch, whereas monolinguals did not.<br><br>**<u>Experiment 3</u>**<br><br>Same as Exp. 1 & 2: Bilinguals demonstrated an advantage in inhibiting old responses and learning new ones compared to monolinguals. |

| | | | | | | | |
|---|---|---|---|---|---|---|---|
| **11**<br><br>**HT** | Liberman, Woodward, Keysar, & Kinzler. (2017). Exposure to multiple languages enhances communication skills in infancy. | 14-17 months | Monolinguals (English) | <u>Languages:</u><br>English<br>+<br>Spanish<br>French<br>Yoruba<br>Filipino<br>Hebrew<br>Hindi<br>(+ 3 additional languages)<br><br><u>Language exposure:</u><br>Range L2: 5 - 70%*<br><br>*Note: 2 infants were exposed to 2 additional languages | - Visual perspective taking | Interactive social communication task requiring infants to take speaker's perspective in order to understand her meaning:<br><br>Infants were asked to hand the experimenter an object, and had to choose between either the object that was visible to both of them or an identical object that only the infant could see. | Multilinguals performed above chance at handing experimenter the correct toy based on her visual perspective; monolinguals were at chance.<br><br>When toys were different, groups performed the same.<br><br>Majority of multilingual infants took experimenter's perspective, not so with monolinguals.<br><br>*Percent exposure to the L2 for multilinguals did not correlate with performance on perspective-taking task. |
| **12**<br><br>**HT** | Poulin-Dubois, Blaye, Coutya, & Bialystok. (2011). The effects of bilingualism on toddlers' executive functioning. | 24 months | Monolinguals (English or French) | <u>Languages:</u><br>L1:<br>English or French<br><br>L2:<br>English<br>French<br>Hebrew<br>Turkish<br>Italian<br><br><u>Language exposure:</u><br>Mean L2 = 35% | Executive function:<br>- Inhibition ("delay and conflict categories of executive function") | <u>Conflict:</u><br>- Multi-location<br><br>- Shape Stroop<br><br>- Reverse Categorization<br><br><u>Delay:</u><br>- Snack delay<br><br>- Gift delay | <u>Shape Stroop</u>: Bilinguals performed better on post-Stroop phase than monolinguals.<br><br>No effect of language group emerged for the other tasks.<br><br>*Significant correlation was found between L2 exposure and proportion of correct Stroop trials. |
| **13**<br><br>**HT** | Singh, Fu, Rahman, Hameed, Sanmugam, Agarwal, Jiang, Chong, Meaney, & Rifkin-Graboi. (2015). Back to basics: A bilingual advantage in infant visual habituation. | Mean = 6.5 months | Monolinguals (English) | <u>Languages:</u><br>English<br>+<br>Chinese<br>Tamil/Hindi/Bengali<br>Malay<br><br><u>Language exposure:</u><br>Mean L2 = 41% | Basic information processing | Visual habituation task | Bilinguals showed advantage in efficiency with which they habituated, and in visual recognition memory for habituated stimulus:<br>- Greater attention decrement & steeper slope during habituation phase<br>- Longer fixation on novel stimulus |
| **14** | Verhagen, de Bree, & Unsworth. | Mean = 24 months | Monolinguals (Dutch) | <u>Languages:</u><br>Dutch<br>+ | Cognitive control:<br>- Inhibitory control<br>- Selective attention | <u>Inhibitory control:</u><br>Spatial conflict task | No significant differences emerged between monolinguals and bilinguals on any of the cognitive control measures. |

| | | | | | | | |
|---|---|---|---|---|---|---|---|
| **HT** | (2020). Effects of bilingual language use and language proficiency on 24-month-olds' cognitive control. | | | English<br>German<br>Italian<br>Spanish<br>Frisian<br>Brazilian Portuguese<br>(+ 7 additional languages)<br><br><u>Language exposure:</u><br>34/37 bilinguals were exposed to both languages at home<br><br>3/37 were exposed to non-Dutch at home and Dutch outside home | - Attention shifting | <u>Selective attention:</u><br>Visual search task<br><br><u>Attention shifting:</u><br>Early Childhood Behavior Questionnaire (ECBQ) to assess attention focusing, inhibitory control, and attention shifting based on parental report | Bilingual children who used both languages very regularly with their parents obtained higher effortful control ratings on the ECBQ compared to children with less regular use of both languages. |
| **PRESCHOOL** | | | | | | | |
| **15**<br><br>**HM** | Aktan-Erciyes. (2020). Longitudinal effects of second language on first language narrative skills and executive functions of preschool children. | **<u>Study 1:</u>**<br><br>4 years<br><br>**<u>Study 2:</u>**<br><br>5 years (same children as Study1) | **<u>Study 1:</u>**<br><br>Monolinguals (Turkish)<br><br>**<u>Study 2:</u>**<br><br>Monolinguals (Turkish) | **<u>Study 1:</u>**<br><br><u>Languages:</u><br>Turkish<br>+<br>English<br><br><u>Language exposure:</u><br>Exposed to exclusively English at school for two years prior to beginning the study; determined to have comparable English proficiency to monolingual English peers by the Peabody Picture Vocabulary Test-4<br><br>**<u>Study 2:</u>**<br><br>Same as Study 1, except now participants had three years of bilingual experience. | **<u>Study 1:</u>**<br><br>Executive function (EF):<br>- Inhibition and switching<br><br>**<u>Study 2:</u>**<br><br>Executive function (EF):<br>- Inhibition and switching | **<u>Study 1:</u>**<br><br>Dimensional Change Card Sort task (DCCS; for inhibition and switching)<br><br>**<u>Study 2:</u>**<br><br>Dimensional Change Card Sort task (DCCS; for inhibition and switching): In this study, experimenter used a more difficult version in which children also had to attend to a border around each card, indicating whether to play the 'colour game' or 'shape game' with the cards | **<u>Study 1:</u>**<br><br>No significant differences emerged between bilingual and monolingual children.<br><br>**<u>Study 2:</u>**<br><br>No significant differences emerged between bilingual and monolingual children. |

| | | | | | | | |
|---|---|---|---|---|---|---|---|
| 16<br><br>**HM** | Bain & Yu. (1980). Cognitive consequences of raising children bilingually: 'One Parent, One Language'. | **Study 1:**<br><br>22-24 months<br><br>**Study 2:**<br><br>Same participants at 46-48 months | **Study 1:**<br><br>Alsace:<br>Monolinguals (German or French)<br><br>Alberta:<br>Monolinguals (English or French)<br><br>Hong Kong:<br>Monolinguals (Chinese or English)<br><br>**Study 2:**<br><br>Same as Study 1 | **Study 1:**<br><br>Alsace Languages:<br>German<br>+<br>French<br><br>Alberta Languages:<br>French<br>+<br>English<br><br>Hong Kong Languages:<br>Chinese<br>+<br>English<br><br>All: Language exposure:<br>At least one parent was bilingual and spoke to the child using a different language than the other parent every day<br><br>**Study 2:**<br><br>Same as Study 1 | **Study 1:**<br><br>- "Voluntary cognitive control"; Stage 1 of cognitive development according to Luria (1961)<br><br>**Study 2:**<br><br>- "Voluntary cognitive control"; Stage 2 of cognitive development according to Luria (1961) | **Study 1:**<br><br>Task in which the child had to locate a marble under a cup, with different levels of delay or an instruction change after repeated trials.<br><br>**Study 2:**<br><br>Task in which the child had to squeeze a ball in response to a certain colour of light, and withhold that response for another colour of light, with different levels of difficulty. | **Study 1:**<br><br>No significant differences emerged between language groups on any of the task variants (but there was a trend toward bilinguals having more correct responses).<br><br>**Study 2:**<br><br>No significant differences emerged between groups on first level of difficulty, but bilinguals outperformed monolinguals on the two higher difficulty levels. |
| 17<br><br>**HT** | Barac, Moreno, & Bialystok. (2016). Behavioral and electrophysiological differences in executive control between monolingual and bilingual children. | 5 years | Monolinguals (English) | Languages:<br>English<br>+<br>Spanish<br>French<br>Mandarin<br>Greek<br>Korean<br>Ukrainian<br>(+ 6 additional languages)<br><br>Language exposure:<br>Spoke 1 language at school, plus another language at home; LSBQ scores showed relatively equal use of both at home; one third were 'crib bilinguals', rest | Executive function:<br>- Response inhibition (RI; simple & complex)<br>- Attention | Gift delay (simple RI)<br><br>Go/No-Go task (complex RI)<br><br>Attention Network Task (ANT; attention) | Gift delay: No significant differences emerged between language groups.<br><br>Go/No-Go: Bilinguals had better overall accuracy on go and no-go trials than monolinguals ($p$ = 0.02).<br>Bilinguals were faster on go trials than monolinguals ($p$ = 0.02).<br>Bilinguals showed better 'perceptual sensitivity' to conditions ($p$ = 0.04).<br><br>ANT: Bilinguals had better overall accuracy on congruent & incongruent trials than monolinguals ($p$ = 0.03).<br>No significant differences on any of the attentional indexes.<br>No significant differences on RTs. |

| | | | | | | | |
|---|---|---|---|---|---|---|---|
| | | | | learned one of the languages first | | | |
| **18**<br><br>**HM** | Bialystok. (1999). Cognitive complexity and attentional control in the bilingual mind. | Young group:<br><br>3;2-4;9 years<br><br>Older group:<br><br>5;0-6;3 years | Monolinguals (English) | Languages:<br>Chinese (home)<br>+<br>English (community/ school)<br><br>Language exposure:<br>Spoke one language at home and one in community and at school<br><br>Proficiency test showed comparable English proficiency to monolinguals | Executive function (EF):<br>- Selective attention (SA)<br>- Inhibitory control (IC) | Moving word task (SA; "understanding of the invariance of symbolic relationships")<br><br>Dimensional Change Card Sort Task (DCCS; IC) | Moving Word: Bilinguals and older children showed advantage compared to monolinguals (significant main effects of both language and age group, no interactions).<br><br>DCCS: On post-switch phase, bilinguals and older children showed an advantage compared to monolinguals. |
| **19**<br><br>**HM** | Bialystok & Martin. (2004). Attention and inhibition in bilingual children: Evidence from the dimensional change card sort task. | **Study 1:**<br><br>5 years<br><br>**Study 2:**<br><br>4;6-5 years<br><br>**Study 3:**<br><br>4 years | **Study 1:**<br><br>Monolinguals (English)<br><br>**Study 2:**<br><br>Monolinguals (English)<br><br>**Study 3:**<br><br>Monolinguals (English) | **Study 1:**<br><br>Languages:<br>Chinese<br>+<br>English<br><br>Language exposure:<br>Spoke one language at home and one at school/in the community<br><br>**Study 2:**<br><br>Languages:<br>French<br>+<br>English<br><br>Language exposure: | **Study 1:**<br><br>Executive function:<br>- Response inhibition (RI)<br>- "Conceptual inhibition" (= "the ability to inhibit attention to a prepotent mental representation"; CI)<br><br>**Study 2:**<br><br>Executive function:<br>- Response inhibition (RI)<br>- Conceptual inhibition (CI)<br><br>**Study 3:**<br><br>Executive function:<br>- Response inhibition (RI) | **Study 1:**<br><br>Dimensional Change Card Sort task (DCCS) with 4 conditions of varying complexity (RI & CI)<br><br>**Study 2:**<br><br>DCCS with 2 of the 4 conditions from Study 1 (RI & CI)<br><br>**Study 3:**<br><br>DCCS with 4 conditions: 2 based on perceptual features, 2 based on semantic features (RI & CI) | **Study 1:**<br><br>Bilinguals outperformed monolinguals on 2 mid-difficulty conditions of DCCS; performance did not significantly differ on easiest or hardest versions of the task.<br><br>Based on pass/fail scoring, bilinguals showed an advantage on only one version of the task.<br><br>**Study 2:**<br><br>Bilinguals outperformed monolinguals on both task versions.<br><br>Based on pass/fail scoring, bilinguals showed advantage on just one.<br><br>**Study 3:** |

| | | | | | | | |
|---|---|---|---|---|---|---|---|
| | | | | Spoke French at school, and English in extra-curricular activities/with friends/ in community<br><br>**<u>Study 3:</u>**<br><br><u>Languages:</u><br>Chinese<br>+<br>English<br><br><u>Language exposure:</u><br>Spoke one language at home and one at school/in the community | - Conceptual inhibition (CI) | | Bilinguals outperformed monolinguals on both perceptual conditions.<br><br>Performance did not differ on semantic conditions.<br><br>Based on pass/fail, bilinguals showed advantage on both perceptual tasks. |
| **20**<br><br>**HM** | Carlson & Meltzoff. (2008). Bilingual experience and executive functioning in young children. | Mean = 6 years | Monolinguals (English)<br><br>Immersion education group (English-speaking learners of Spanish or Japanese with 6 months of L2 exposure) | <u>Languages:</u><br>Spanish<br>+<br>English<br><br><u>Language exposure:</u><br>Spoke a mixture of both languages in the home | Executive function:<br>- Conflict (inhibition with higher working memory demands)<br>- Delay (inhibition with low working memory demands) | <u>Conflict tasks</u>:<br>Dimensional Change Card Sort task (DCCS)<br><br>Simon Says task<br><br>Visually cued recall<br><br>Kansas Reflection/Impulsivity Scale (KRISP)<br><br>Comprehensive Test of Nonverbal Intelligence (C-TONI)<br><br>Attention Network Task (ANT)<br><br><u>Delay tasks</u>:<br>Delay of Gratification task<br><br>Statue task<br><br>Gift delay with cover | <u>Composite EF score:</u><br><br>Bilinguals significantly outperformed monolingual and immersion groups (*ps* < .01) based on composite score.<br><br><u>Individual tasks:</u><br><br>Bilinguals outperformed immersion group on Visually Cued Recall ($p < .05$).<br><br>Bilinguals outperformed monolinguals on the DCCS ($p < .05$).<br><br><u>Conflict vs. Delay:</u><br><br>Performance on conflict tasks was significantly related to language group, with bilinguals outperforming immersion and monolingual groups (*ps* < .01).<br><br>Performance on delay tasks was unrelated to language group. |
| **21**<br><br>**HM** | Crespo & Kaushanskaya. (2021). Is 10 better than 1? The effect of speaker variability on children's cross- | 4-7 years | Monolinguals (English) | <u>Languages:</u><br>Spanish<br>+<br>English<br><br><u>Language exposure:</u><br>Mean weekly exposure to English: | Sustained attention | Conner's Kiddie Continuous Performance Test – Second Edition | No significant differences between monolinguals and bilinguals emerged. |

| | | | | | | | |
|---|---|---|---|---|---|---|---|
| | situational word learning. | | | 44.76%<br>Mean weekly exposure to Spanish: 41.82% | | | |
| **22**<br><br>**HM** | Darcy. (1946). The effect of bilingualism upon the measurement of the intelligence of children of preschool age. | 2;6-4;6 years | Monolinguals (English) | Languages:<br>Italian (home)<br>+<br>English (outside home)<br><br>Language exposure:<br>Heard & spoke one language at home "always or most of the time" and heard and spoke the other language outside the home "always or most of the time" | - Verbal and non-verbal intelligence (IQ)<br><br>- Mental age (MA) | Verbal intelligence + MA:<br>1937 Revision of the Stanford Binet Scale, Form L<br><br>Non-verbal intelligence + MA:<br>Atkins Object-fitting Test, Form A | IQ:<br>*Stanford Binet* (verbal):<br>Monolinguals had significantly higher IQ than bilinguals.<br><br>*Atkins* (non-verbal):<br>Bilinguals had significantly higher IQ than monolinguals.<br><br>Mental age:<br>*Stanford Binet* (verbal):<br>Monolinguals had higher MA than bilinguals, but did not reach significance.<br><br>*Atkins* (non-verbal):<br>Bilinguals had significantly higher MA than monolinguals. |
| **23**<br><br>**HM** | Diaz & Farrar. (2018a). Do bilingual and monolingual preschoolers acquire false belief understanding similarly? The role of executive functioning and language. | 3;4 - 5;5 years | Monolinguals (English) | Languages:<br>English<br>+<br>Spanish<br><br>Language exposure:<br>61% exposed to both languages since birth; parents reported that children were fluent in both and interacted regularly with speakers of both | Theory of mind (ToM):<br>- False belief reasoning (FB)<br><br>Executive function:<br>- Inhibitory control (IC)<br>- Cognitive flexibility (CF) | FB tasks:<br>Unexpected contents task<br><br>Unexpected location task<br><br>Object disappearance<br><br>Appearance-reality, object identity task<br><br>Appearance-reality, object property task<br><br>EF tasks:<br>Day/Night Stroop-like task (IC)<br><br>Bear/Dragon Simon Says task (IC)<br><br>Dimensional Change Card Sort task (DCCS; CF) | FB composite:<br>After controlling for differences in language ability, bilinguals outperformed monolinguals ($p$=0.49).<br><br>EF composite:<br>After controlling for language ability, marginally significant advantage for bilinguals ($p$=0.06). |

| | | | | | | | |
|---|---|---|---|---|---|---|---|
| **24**<br><br>**HM** | Diaz & Farrar. (2018b). The missing explanation of the false-belief advantage in bilingual children: A longitudinal study. | 3-5;6 years | Monolinguals (English) | Languages:<br>English<br>+<br>Spanish<br><br>Language exposure:<br>77.5% exposed to both languages since birth; parents reported that children were fluent in both languages and interacted regularly with speakers of both | Theory of mind (ToM):<br>- False belief reasoning (FB)<br><br>Executive function:<br>- Inhibitory control (IC)<br><br>- Cognitive flexibility (CF) | FB tasks:<br>Unexpected contents task<br><br>Unexpected location task<br><br>Appearance-reality, object identity task<br><br>EF tasks:<br>Bear/Dragon Simon Says task (IC)<br><br>Happy-Sad Stroop-like task (IC)<br><br>Dimensional Change Card Sort task (DCCS; CF) | Time 1:<br>FB: Once significant differences in receptive vocabulary were controlled, bilinguals showed an advantage on FB reasoning composite score.<br><br>EF: Once differences in receptive vocabulary were controlled, bilinguals outperformed monolinguals on EF composite score, DCCS, and Bear/Dragon task.<br><br>No significant difference between monolinguals and bilinguals on Happy-Sad Stroop.<br><br>Time 2:<br>FB: No differences between groups.<br><br>EF: No differences between groups. |
| **25**<br><br>**HT** | Dicataldo & Roch. (2020). Are the Effects of Variation in Quantity of Daily Bilingual Exposure and Socioeconomic Status on Language and Cognitive Abilities Independent in Preschool Children? | 3;8-6;3 years | Continuum of bilingualism indexed by 2 variables:<br><br>1) Length of exposure to “language of context”, i.e. Italian (LELC): computed as the difference between age of L2 Italian acquisition and time of testing<br><br>Range = 24-75 months for participants with an L2 (n=28)<br><br>2) Daily exposure to language of context (DELC): mean percentage of daily use of Italian<br><br>Range = 29-100% | Languages:<br>Italian<br>+<br>Romanian<br>Chinese<br>Arabic<br>Moldavian<br>Russian<br>Albanese<br>Turkish | Executive function:<br>- Working memory (WM)<br>- Inhibition (I)<br>- Attention-shifting (AS)<br><br>Theory of Mind (ToM):<br>- False belief (FB) reasoning | EF:<br>Forward and Backward Digit Span (WM)<br><br>Day/Night Stroop task (I)<br><br>Dimensional Change Card Sort (DCCS; AS)<br><br>FB Reasoning:<br>Unexpected contents task | (Conducted hierarchical regression analyses)<br><br>EF:<br>Variation in LELC independently accounted for<br>- 18% of variance in WM<br>- 7% of variance in Inhibition<br>- 10% of variance in attention-shifting<br><br>Variation in DELC independently accounted for<br>- 3% of variance in WM<br><br>ToM:<br>Variation in LELC did not significantly account for variance in FB reasoning.<br><br>Significant interaction between variation in SES and variation in DELC whereby children with higher SES and higher DELC performed better on FB task.<br><br>DELC did not account significantly for variance in ToM independently.<br><br>**Authors concluded that their data did not support the idea of a bilingual advantage for any ability other than WM.** |

| | | | | | | | |
|---|---|---|---|---|---|---|---|
| **26**<br><br>**HT** | Haft, Kepinska, Caballero, Carreiras, & Hoeft. (2019). Attentional fluctuations, cognitive flexibility, and bilingualism in kindergarteners. | Mean = 5.68 years | Continuum of L2 exposure from 0-7 years | Languages:<br>English<br>+<br>Spanish<br>Cantonese<br>Mandarin<br>Arabic<br>French<br>Ilocano<br>Other<br><br>Language exposure:<br>24 participants were simultaneous bilinguals<br><br>16 participants had no exposure to a second language (English monolinguals)<br><br>Rest of sample had intermediate levels of L2 exposure | Executive function:<br>- Cognitive flexibility (CF)<br>- Attentional fluctuations (AF) | CF:<br>Dimensional Change Card Sort task (DCCS)<br><br>AF:<br>Tasks of executive control (TEC) – computerized task that combines *N*-back & Go/No-Go paradigms | L2 exposure was not a significant predictor of either CF or AF performance when entered as a continuous variable.<br><br>Group comparisons of children with no L2 exposure and simultaneous bilinguals yielded no significant differences for either CF or AF. |
| **27**<br><br>**HM** | Goetz. (2003). The Effects of bilingualism on theory of mind development. | 3-4 years | Monolinguals (English)<br>+<br>Monolinguals (Mandarin Chinese) | Languages:<br>Mandarin Chinese*<br>+<br>English<br><br>*Some bilinguals had exposure to other dialects of Chinese as well, or to Taiwanese<br><br>Language exposure:<br>Mandarin as primary home language, spoke English outside of the home<br><br>Balanced proficiency according to Peabody Picture Vocabulary Test | Theory of Mind (ToM) | Appearance-Reality task<br>2 versions:<br>- V1: Pen that resembled fish<br>- V2: Sponge that resembled rock<br><br>Level 2 Perspective-Taking task<br>2 versions:<br>- V1: Turtle<br>- V2: Elephant<br><br>False belief (FB) tasks:<br>Unexpected Contents<br>2 versions:<br>- V1: M&Ms box containing toy car<br>- V2: Crayon box containing chocolate bar<br><br>Unexpected Location<br>2 versions:<br>- V1: Two small figures, John and mother, two different coloured drawers<br>- V2: Two dolls, Lily and sister, two different coloured pails | Composite ToM scores (ToM1 for 1st versions, ToM2 for 2nd versions):<br>- Bilinguals & older children performed better than monolinguals & younger children<br>- Bilinguals performed significantly better than monolinguals on Version 1, and nearly significantly better on Version 2<br><br>Individual tasks:<br>- Bilinguals performed significantly better than Chinese monolinguals ($p < 0.05$) on Appearance-Reality Version 1, and marginally better than English monolinguals ($p = 0.053$)<br>- Bilinguals outperformed Chinese monolinguals only on perspective-taking Version 1 ($p < 0.05$)<br>- Bilinguals outperformed all monolinguals on FB Version 2 ($p < 0.05$) |

| | | | | | | | |
|---|---|---|---|---|---|---|---|
| **28**<br><br>**HT** | Goldman, Negen, & Sarnecka. (2014). Are bilingual children better at ignoring perceptually misleading information? A novel test. | Mean = 4;9 years | Monolinguals (English) | <u>Languages:</u><br>English<br>+<br>Chinese<br>Mandarin<br>Spanish<br>Hindi<br>Tamil<br>Cantonese<br>(+ 10 additional languages)<br><br><u>Language exposure:</u><br>All children attended English-language daycare<br><br>Two subgroups:<br>1) Children exposed to English + another language at home<br><br>2) Children exposed to only a non-English language at home | Executive function:<br>- Inhibitory control with interference | Non-symbolic numerical discrimination (NSND) task:<br><br>- Participants had to determine which of two cards had more dots on them<br>- Congruent trials: dots on each card were the same size<br>- Incongruent trials: dots were different sizes (e.g. the card w fewer dots had larger ones, and vice versa) | No significant differences emerged between monolinguals and bilinguals in any analyses.<br><br>Percentage of each language spoken at home was not a significant predictor in any regressions. |
| **29**<br><br>**HM** | Gordon. (2016). High proficiency across two languages is related to better mental state reasoning for bilingual children. | 4;6 years | Monolinguals (English) | <u>Languages:</u><br>English<br>+<br>Spanish<br><br><u>Language exposure:</u><br>Most had parents who spoke Spanish, or Spanish & English, in the home.<br><br>Similar proficiency in both according to Peabody Picture Vocabulary Test (English and Spanish versions). | "Mental state reasoning" | Diverse desires task<br><br>Diverse beliefs task<br><br>Knowledge access task<br><br>Contents false belief task<br><br>Explicit false belief task<br><br>Belief-emotion task<br><br>Real-apparent emotions task | <u>Composite mental state scores</u>: No significant difference between monolinguals and bilinguals.<br><br><u>Diverse Desires</u>:<br>Bilinguals scored higher than monolinguals.<br><br><u>Explicit FB</u>:<br>Monolinguals scored higher than bilinguals.<br><br>No other group differences emerged on individual tasks.<br><br>English proficiency predicted mental state task performance for monolinguals but not for bilinguals. |
| **30**<br><br>**HM** | Kovacs. (2009). Early bilingualism enhances mechanisms of false-belief | 3 years | Monolinguals (Romanian) | <u>Languages:</u><br>Romanian<br>+<br>Hungarian<br><br><u>Language exposure:</u> | Theory of Mind (ToM):<br>- False belief reasoning (FB) | Standard ToM FB task (ToM)<br><br>Modified ToM FB task (MToM) depicting a language-switch situation leading to different belief attributions for | Bilinguals performed better on both ToM tasks than monolinguals (ToM: *p*=0.01; MToM: *p*=0.03), and performed equally well on both versions.<br><br>Monolinguals did not differ in their |

| | | | | | | | |
|---|---|---|---|---|---|---|---|
| | reasoning. | | | Had "parents of different mother tongues who each address the child in their native language", and daily exposure to both languages | | monolingual vs. bilingual children | performance on the two tasks. |
| **31**<br><br>**HM** | Leikin & Tovli. (2014). Bilingualism and creativity in early childhood. | Mean = 6 years | Monolinguals (Hebrew) | <u>Languages:</u><br>Hebrew<br>+<br>Russian<br><br><u>Language exposure:</u><br>Spoke one language dominantly at home and one at school/in community; raters of each language rated children's proficiency in both languages as being equal and comparable to that of the monolinguals for their L2; bilinguals were born in the L2 environment | General creative thinking<br><br>Working memory (WM) | <u>Creativity:</u><br>Creating Equal Number (CEN) task:<br>- Children have to come up with as many ways to make the number of bottle caps on either side of a table equal, by moving them around<br><br><u>WM:</u><br>Listening span task | <u>Creativity:</u><br>Groups did not differ on number of solutions given (fluency score).<br><br>Bilingual children gave answers that scored higher on flexibility and originality than monolingual children.<br><br><u>WM:</u><br>Bilingual children remembered significantly more words than monolingual children ($p$ = .033). |
| **32**<br><br>**HM** | Mehrani & Zabihi. (2017). A comparative study of shifting ability, inhibitory control and working memory in monolingual and bilingual children. | 4;6 years | Monolinguals (Persian) | <u>Languages:</u><br>Persian<br>+<br>Turkish<br><br><u>Language exposure:</u><br>Evaluated as having similar functional proficiency in their L1/L2; used both at home and school; parents spoke either one of each language, or both | Executive function:<br>- Inhibitory control (IC)<br><br>- Set shifting (SS)<br><br>Working memory (WM) | <u>Inhibitory control:</u><br>Simon task<br><br><u>Set shifting:</u><br>Dimensional change card sort (DCCS)<br><br><u>WM:</u><br>Forward + Backward Digit recall tasks | Bilinguals outperformed monolinguals on DCCS ($p < 0.001$) and Simon task ($p < 0.001$).<br><br>No significant difference between groups on WM tasks. |
| **33**<br><br>**HM** | Namazi & Thordardottir. (2010). A working memory, not bilingual advantage, in controlled | 5 years | Monolinguals (French)<br>+<br>Monolinguals (English) | <u>Languages:</u><br>English<br>+<br>French<br><br><u>Language exposure:</u><br>40-60% daily exp. to both languages before | Verbal + visual working memory (WM)<br><br>Visual controlled attention (CA) | <u>Verbal WM:</u><br>Listening span task<br><br><u>Visual WM:</u><br>Pattern recall task<br><br><u>Visual CA:</u><br>Simon task | <u>Verbal WM</u>: No significant differences were found between monolinguals and bilinguals.<br><br><u>Visual WM</u>: No significant differences.<br><br><u>CA</u>: No significant differences. |

| | | | | | | | |
|---|---|---|---|---|---|---|---|
| | attention. | | | 3 years | | | *Found correlations between performance on pattern recall and Simon task, but was unrelated to language group. |
| **34**<br><br>**HM** | Nguyen & Astington. (2014). Reassessing the bilingual advantage in theory of mind and its cognitive underpinnings. | 3-5 years | Monolinguals (English)<br>+<br>Monolinguals (French) | Languages:<br>English<br>+<br>French<br><br>Language exposure:<br>Exposed from before 8 months to L1/L2; exposed to each language a min. 30% of the time | Theory of Mind (ToM)<br><br>Executive function (EF)<br>- Conflict inhibition<br><br>Working memory (WM) | ToM:<br>False belief (FB) tasks:<br>Unexpected-location task<br><br>Unexpected-contents task<br><br>EF:<br>Stroop task<br><br>WM:<br>Backward Word Span (BWS) | Without controlling for age or verbal ability:<br>No significant difference between bilinguals and monolinguals on any cognitive task.<br><br>Controlling for age and verbal ability:<br>Bilinguals outperformed monolinguals on FB tasks and BWS task, but not Stroop task. |
| **35**<br><br>**HM** | Tran, Arredondo, & Yoshida. (2015). Differential effects of bilingualism and culture on early attention: A longitudinal study in the U.S., Argentina, and Vietnam. | Longitudinal design:<br>Tested at 5 time points from 3-5 years of age | United States:<br>Monolinguals (English)<br><br>Vietnam:<br>Monolinguals (Vietnamese)<br><br>Argentina:<br>Monolinguals (Spanish) | US Languages:<br>English<br>+<br>Spanish<br>Vietnamese<br><br>Vietnam Languages:<br>Vietnamese<br>+<br>Cantonese<br><br>Language exposure:<br>Total vocabulary in both languages fell above 20th percentile on MacArthur Communicative Development Inventory; conceptual vocabulary not different from other language. groups<br><br>*No bilinguals recruited in Argentina because they could not be matched for SES with Argentinian monolinguals | Executive control:<br>- Attention | Attention Network Test (ANT) | Bilinguals outperformed monolinguals on accuracy across all time points, and outperformed them on RT at Time 2-5.<br><br>*Note: Bilingual advantage was persistent, but modulated by culture group. |
| **36** | Tran, Arredondo, & Yoshida. (2019). Early executive | Longitudinal design:<br>Tested at ages 3, 3;6, and 4 | United States:<br>Monolinguals (English) | US Languages:<br>English<br>+<br>Spanish | Executive function:<br>- Selective attention (SA)<br>- Switching<br>- Inhibition | Dimensional Change Card Sort task (DCCS; for SA, switching, inhibition, and monitoring) | DCCS:<br><br>Bilinguals outperformed monolinguals overall across time points, but |

| | | | | | | | |
|---|---|---|---|---|---|---|---|
| **HM** | function: The influence of culture and bilingualism. | years | <u>Vietnam:</u><br>Monolinguals (Vietnamese)<br><br><u>Argentina:</u><br>Monolinguals (Spanish) | Vietnamese<br><br><u>Language exposure:</u><br>Regularly exposed to both languages at home, and English out of the home<br><br><u>Vietnam Languages:</u><br>Vietnamese<br>+<br>Cantonese<br><br><u>Language exposure:</u><br>Regularly exposed to both languages at home, and Vietnamese outside the home<br><br>*No bilinguals recruited in Argentina because they could not be matched for SES with Argentinian monolinguals | - Monitoring<br>- Response inhibition (RI) | Day/Night task (for RI)<br><br>Bear/Dragon Simon Says task (for RI)<br><br>Gift Delay task (for RI) | particularly at Time 1, all bilingual groups outperformed all monolingual groups.<br><br><u>Day/Night:</u><br><br>Bilinguals outperformed monolinguals overall; in particular, bilinguals from Eastern cultures (Viet-English and Viet-Cantonese) outperformed other groups across time points.<br><br>Moderated by culture: Eastern bilinguals outperformed non-Eastern monolinguals, but NOT Eastern monolinguals.<br><br><u>Bear/Dragon:</u><br><br>No significant effects of language status.<br><br><u>Gift Delay:</u><br><br>Bilinguals outperformed monolinguals overall with no significant interaction with Time. |
| **37**<br><br>**HT** | Yoshida, Tran, Benitez, & Kuwabara. (2010). Attentional control and early word learning. | 3 years | Monolinguals (English) | <u>Languages:</u><br>English<br>+<br>Spanish<br>Bengali<br>Chinese<br>Russian<br>Urdu<br>Vietnamese<br><br><u>Language exposure:</u><br>Primarily spoke non-English language at home | Executive function:<br>- Attentional control | Attention Network Task (ANT) | Bilinguals outperformed monolinguals (more correct responses; $p < 0.05$). |
| **38**<br><br>**HT** | Yoshida, Tran, Benitez, & Kuwabara. (2011). Inhibition and adjective learning in bilingual and monolingual | 3 years | Monolinguals (English) | <u>Languages:</u><br>English<br>+<br>Spanish<br>Chinese<br>French<br>Russian<br>Urdu<br>Vietnamese | Executive function:<br>- Attentional control | Attention Network Task (ANT) | Bilinguals were more accurate on ANT ($p < 0.01$) than monolinguals.<br><br>Bilinguals were also faster, but the difference was not significant ($p = 0.25$). |

| | | | | | | | |
|---|---|---|---|---|---|---|---|
| | children. | | | Language exposure:<br>Eng: Mean = 7.45 hours daily exp.<br><br>Non-Eng: Mean = 6.73 hours daily exp. | | | |
| | | | | **SCHOOL** | | | |
| **39**<br><br>**HM** | Andreou, Tsimpli, Masoura, & Agathapoulou. (2021). Cognitive mechanisms of monolingual and bilingual children in monoliterate educational settings: Evidence from sentence repetition. | 8-12 years | Monolinguals (Greek) | Languages:<br>Albanian<br>+<br>Greek<br><br>Language exposure:<br>Exposed to 63.4% Greek and 36.6% Albanian from birth to age 6; used Greek more than Albanian | Non-verbal intelligence<br><br>Verbal working memory (VWM)<br><br>Visuospatial working memory (VSWM)<br><br>Updating | Non-verbal IQ:<br>Raven's Progressive Colored Matrices<br><br>VWM:<br>Backwards Digit Recall task<br><br>VSWM:<br>Rotating Figure task<br><br>Updating:<br>2-Back task | Non-verbal IQ & VWM:<br>No significant differences between language groups emerged.<br><br>VSWM & Updating:<br>Monolinguals outperformed bilinguals. |
| **40**<br><br>**HM** | Anton, Dunabeitia, Estevez, Hernandez, Castillo, Fuentes, & Carreiras. (2014). Is there a bilingual advantage in the ANT task? Evidence from children. | Group 1:<br>Mean = 7.5 years<br><br>Group 2:<br>Mean = 9.5 years<br><br>Group 3:<br>Mean = 11.4 years | Monolinguals (Spanish) | Languages:<br>Spanish<br>+<br>Basque<br><br>Language exposure:<br>Exposed to both languages daily in school – average proficiency was lower in L2 | Executive function:<br>- Attentional control | Child Attentional Network Task (ANT) | General RTs and error rates: No significant main effects of language group or interactions emerged.<br><br>No main effects/interactions with language group emerged for Conflict effect, Alerting effect, Orienting effect, or Validity effect. |

| | | | | | | | |
|---|---|---|---|---|---|---|---|
| **41**<br><br>**HT** | Bialystok & Viswanathan. (2009). Components of executive control with advantages for bilingual children in two cultures. | 8 years | Monolinguals (English) | Canada Languages:<br>English<br>+<br>Cantonese<br>Croatian<br>French<br>Hebrew<br>Hindi<br>Kannada<br>(+ 7 additional languages)<br><br>India Languages:<br>English<br>+<br>Tamil<br>Telugu<br><br>Language exposure:<br>Participants spoke English at school and other language at home; had spoken both since birth | Executive control:<br>- Response suppression (RS)<br>- Inhibitory control (IC)<br>- Switching | 'Faces task':<br>- Participants responded to an asterisk on a screen according to rules based on the direction and colour of the eyes in a face – assessed RS, IC, and switching | Straight-eyes condition: Bilinguals were faster than monolinguals on green-eye, red-eye, and mixed presentation trials.<br><br>Gaze-shift condition: Bilinguals were faster than monolinguals, no interactions emerged.<br><br>Bilingual groups had significantly lower RT costs from inhibition manipulation and switching manipulation than monolinguals. |
| **42**<br><br>**HM** | Bosma, Hoekstra, Versloot, & Blom. (2017). The Minimal and short-lived effects of minority language exposure on the executive functions of Frisian-Dutch bilingual children. | Time 1: 5-6 years<br><br>Time 2: 6-7 years<br><br>Time 3: 7-8 years | Low degree of bilingualism (DoB), quantified by daily exposure and language balance | Languages:<br>Dutch<br>+<br>West Frisian<br><br>Language exposure:<br>High degree of bilingualism (DoB), quantified by daily exposure and language balance | Executive function (EF):<br>- Selective attention (SA)<br>- Interference suppression (IS)<br><br>Working memory (WM):<br>- Verbal WM<br>- Visuospatial WM | EF:<br>Sky Search task (SA)<br><br>Flanker task (IS)<br><br>WM:<br>Backward Digit Span task (Verbal)<br><br>Backward Dot Matrix task (Visuospatial) | The only cognitive measure that was significantly predicted by degree of bilingualism was the Sky Search task.<br><br>DoB predicted (better) SS performance at T1, but not T2 or T3.<br><br>Intensity of exposure to Frisian at home predicted better SS performance. |
| **43**<br><br>**HT** | Cockcroft. (2016). A comparison between verbal working memory and vocabulary in bilingual and monolingual South African school beginners: | Mean = 6.73 years | Monolinguals (English) | Languages:<br>English<br>+<br>isiXhosa<br>isiZulu<br><br>Language exposure:<br>Spoke both language daily (English in school, other language | Non-verbal intelligence<br><br>Verbal working memory (VWM) | Non-verbal IQ:<br>Raven's Coloured Progressive Matrices<br><br>VWM:<br>4 subtests from the Automated Working Memory Assessment:<br><br>Digit recall | Non-verbal IQ:<br>Monolinguals outperformed bilinguals (*p*=.024).<br><br>VWM:<br>No significant differences between groups emerged, even when controlling for vocabulary and non-verbal IQ. |

| | | | | | | | |
|---|---|---|---|---|---|---|---|
| | Implications for bilingual language assessment. | | | at home) | | Non-word recall<br><br>Counting recall<br><br>Backward digit recall | |
| **44**<br><br>**HM** | Crespo, Gross, & Kaushanskaya. (2019). The effects of dual language exposure on executive function in Spanish-English bilingual children with different language abilities. | 5-11 years | Graded dual-language exposure from low to high | <u>Languages:</u><br>English<br>+<br>Spanish<br><br>3 groups:<br>1) Simultaneous bilinguals<br>2) Spanish L1<br>3) English L1<br><br><u>Language exposure:</u><br>Assessed amount of dual-language (DL) exposure based on parent report of daily time spent in contexts where both languages were used | Executive function (EF):<br>- Inhibitory control<br>- Shifting<br>- Monitoring | <u>EF:</u><br>Dimensional Change Card Sort Task (DCCS) | Group membership did not significantly predict performance (*p*=.84).<br><br>Significant interaction between DL exposure and language skills such that children showed smaller shifting costs with higher DL exposure as their language skills increased (*p*=.03).<br><br>No significant interaction between DL exposure and language skills for switching costs.<br><br>Significant interaction between DL exposure and language skills such that children showed smaller mixing costs with higher DL exposure as their language skills increased (*p*=.001).<br><br>Main effects for DL exposure alone were never significant. |
| **45**<br><br>**HT** | Czapka, Wotschack, Klassert, & Festman. (2020). A path to the bilingual advantage: Pairwise matching of individuals. | Monolinguals<br>Mean age = 8.86 years<br><br>Multilinguals<br>Mean age = 8.97 years | Monolinguals (German) | Multilinguals: 18 bilinguals and 3 trilinguals<br><br><u>Languages:</u><br>German<br>+<br>Turkish<br>Albanian<br>Arabic<br>Bosnian<br>Chinese<br>English<br>(+ 7 additional languages)<br><br><u>Language exposure:</u><br>Spoke German outside the home.<br>In the home:<br>- 24% spoke predominantly languages other than | Executive function:<br>- Interference inhibition<br>- Response inhibition<br>- Updating | <u>Interference inhibition</u>:<br>Bivalent Shape Task (BST)<br><br><u>Response Inhibition</u>:<br>Go/No Go task<br><br><u>Updating</u>:<br>N-back task (1-back followed by 2-back) | <u>BST:</u><br><br>Multilinguals responded faster overall; no significant interactions emerged.<br><br><u>Go/No Go:</u><br><br>Multilingualism did not influence RTs.<br><br><u>N-back:</u><br><br>No significant differences emerged between groups. |

| | | | | | | | |
|---|---|---|---|---|---|---|---|
| | | | | German<br>- 33% spoke mainly German<br>- 24% spoke German and another language similarly often | | | |
| **46**<br><br>**HT** | Engel de Abreu. (2011). Working memory in multilingual children: Is there a bilingual effect? | Mean = 6 years at Time 1<br><br>Tested again 1 & 2 years later | Monolinguals (Luxembourgish) | Languages:<br>Luxembourgish<br>+<br>French<br>Spanish<br>German<br>Dutch<br>Portuguese<br>Czech<br>Italian<br><br>Language exposure:<br>One parent spoke L1, other spoke L2; children were exposed to both on a regular basis, though dominant in Luxembourgish | Verbal working memory (VWM) | Counting Recall task<br><br>Backward Digit Recall task<br><br>Forward Digit Recall task<br><br>Non-Word Repetition task | No significant differences between language groups on Counting Recall, BW Digit Recall, or FW Digit Recall tasks.<br><br>Monolinguals outperformed bilinguals on Non-Word Repetition (*p*=.03). |
| **47**<br><br>**HM** | Engel de Abreu, Cruz-Santos, Tourinho, Martin, & Bialystok. (2012). Bilingualism enriches the poor: Enhanced cognitive control in low-income minority children. | Mean age = 8 years | Monolinguals (Portuguese) | Languages:<br>Portuguese<br>+<br>Luxembourgish<br><br>Language exposure:<br>Used both languages daily, one at home and one in school/community | Abstract reasoning<br><br>Working memory (WM)<br><br>Cognitive control:<br>- Selective attention<br>- Interference suppression | Reasoning:<br>- Raven's Coloured Progressive Matrices<br><br>WM:<br>- Odd-One-Out<br><br>- Dot Matrix<br><br>Cognitive Control:<br>- Sky Search Task<br><br>- Flanker Task | No significant differences emerged between groups on reasoning or WM tasks.<br><br>Bilinguals were faster than monolinguals on Sky Search attention score (controlled for motor speed).<br><br>No differences emerged for the Flanker task accuracy, but bilinguals were faster overall.<br><br>Following principal component analysis to examine whether tasks captured different aspects of EF, bilinguals outperformed monolinguals on 'control' factor (cognitive control measures loaded on this factor), but groups did not differ on 'representation' factor (WM and reasoning measures). |

| | | | | | | | |
|---|---|---|---|---|---|---|---|
| **48**<br><br>**HM** | Ladas, Carroll, & Vivas. (2015). Attentional processes in low-socioeconomic status bilingual children: Are they modulated by the amount of bilingual experience? | **Experiment 1:**<br><br>6-12 years<br><br>**Experiment 2:**<br><br>6-8 years | **Experiment 1:**<br><br>Monolinguals (Greek)<br><br>**Experiment 2:**<br><br>Same characteristics as Exp. 1 | **Experiment 1:**<br><br>Languages:<br>Albanian<br>+<br>Greek<br><br>Language exposure:<br>Used L1/L2 approx. equally (one in home and one at school/in community); high proficiency in both<br><br>**Experiment 2:**<br><br>Same characteristics as Exp. 1 | **Experiment 1:**<br><br>Executive function:<br>- Attention<br><br>**Experiment 2:**<br><br>Executive function:<br>- Attention | **Experiment 1:**<br><br>Child Attentional Network Task<br><br>**Experiment 2:**<br><br>Same task, but different alerting cue (tone rather than asterisk) | **Experiment 1:**<br><br>No significant differences emerged between monolinguals and bilinguals.<br><br>**Experiment 2:**<br><br>No significant differences emerged between monolinguals and bilinguals. |
| **49**<br><br>**HT** | Morales, Calvo, &<br>Bialystok. (2013). Working memory development in monolingual and bilingual children. | **Study 1:**<br>Mean = 5 years<br><br>**Study 2:**<br><br>Two groups:<br>5-year-olds from Study 1<br>+<br>New group of 7-year-olds | **Study 1:**<br>Monolinguals (English)<br><br>**Study 2:**<br>Monolinguals (English | **Study 1:**<br><br>Languages:<br>English<br>+<br>Arabic<br>Bulgarian<br>Cantonese<br>Chinese (dialect unspecified)<br>French<br>Hebrew<br>(+ 7 additional languages)<br><br>Language exposure:<br>Spoke English at school/in community and other lang. at home; used both daily<br><br>**Study 2:**<br><br>Languages:<br>English<br>+<br>Arabic<br>Bengali<br>Cantonese<br>Chinese (dialect unspecified)<br>Farsi<br>Hindi | **Study 1:**<br>Executive control (EC):<br>- Inhibition<br><br>Working memory (WM)<br><br>**Study 2:**<br>Executive control (EC):<br>- Inhibition<br><br>Working memory (WM) | **Study 1:**<br><br>EC + WM:<br>'Pictures task' – Simon-type task<br>- Two levels of WM demand (2 vs. 4 stimuli to respond to)<br>- Two conflict levels (central vs. congruent/incongruent side presentations of stimuli)<br><br>**Study 2:**<br><br>EC + WM:<br>Frog Matrices Task (FMT) – Variant of Corsi blocks task | **Study 1:**<br><br>For conflict levels, bilinguals' accuracy was not reduced in incongruent trials, but it was for monolinguals.<br><br>No difference between groups emerged when comparing accuracy on two WM levels.<br><br>Bilinguals had generally faster responses on both WM and conflict levels.<br><br>**Study 2:**<br><br>No differences emerged between bilinguals and monolinguals on analysis of memory span in either task condition.<br><br>Analysis of proportion of correct responses showed higher scores for bilinguals.<br><br>Younger bilinguals outperformed younger monolinguals on simultaneous condition.<br><br>Older bilinguals had an advantage over younger ones in the sequential condition. |

| | | | | | | | |
|---|---|---|---|---|---|---|---|
| | | | | (+ 11 additional languages)<br><br>Language exposure:<br>Same exposure characteristics as Study 1 | | | |
| **50**<br><br>**HM** | Park, Ellis Weismer, & Kaushanskaya. (2018). Changes in executive function over time in bilingual and monolingual school-aged children. | Year 1: 9 years<br><br>Year 2: 10 years | Monolinguals (English) | Languages:<br>Spanish<br>+<br>English<br><br>Language exposure:<br>Mean Eng. = 56%<br>Mean Span. = 44%<br><br>Attended school in English. | Executive function:<br>- Inhibition<br>- Updating<br>- Task shifting | Inhibition:<br>Flanker task<br><br>Updating:<br>Corsi blocks test<br><br>Task shifting:<br>Dimensional Change Card Sort task (DCCS) | Inhibition:<br>Yr. 1: No significant differences between groups.<br>Yr. 2: Bilinguals outperformed monolinguals (*p*=.038).<br><br>Updating:<br>Yr. 1 & 2: No main effect of language group or interaction.<br><br>Task shifting:<br>Bilinguals were faster than monolinguals for non-switch trials in mixed condition (*p*=.039).<br><br>Bilinguals showed smaller mixing costs than monolinguals in both years (*p*=.009 & *p*=.011). |
| **51**<br><br>**HT** | Pino Escobar, Kalashnikova, & Escudero. (2018). Vocabulary matters! The relationship between verbal fluency and measures of inhibitory control in monolingual and bilingual children. | M=7;10 years | Monolinguals (English) | Languages:<br>English<br>+<br>Arabic<br>Spanish<br>Cantonese<br>Mandarin<br>Malay<br>Russian<br>(+ 4 additional languages)<br><br>Language exposure<br>Mean L2 = 34.12% | Inhibitory control | Dimensional change card sort task (DCCS)<br><br>Day-Night Stroop task | No significant differences were found between monolinguals and bilinguals for either task. |
| **52**<br><br>**HT** | Poarch. (2018). Multilingual language control and executive function: A replication study. | 5-13 years | Monolingual (German) learners of English as L2 | *n=34 bilinguals, n=48 trilinguals<br><br>Bilingual Languages:<br>German<br>+<br>English<br><br>Language exposure: | Executive function:<br>- Conflict monitoring and inhibitory control | Simon task<br><br>Flanker task | *Bi- and trilinguals were analyzed together as 1 group of multilinguals<br><br>Simon task:<br>No significant differences between multilinguals and monolinguals emerged.<br><br>Flanker task:<br>Multilinguals resolved conflict significantly faster than monolinguals |

| | | | | | | | |
|---|---|---|---|---|---|---|---|
| | | | | Lived in homes where both languages were primary languages; both languages used at school<br><br><u>Trilingual Languages:</u><br>German<br>+<br>Arabic<br>Croatian<br>Danish<br>Dutch<br>Eritrean<br>Greek<br>(+ 14 additional languages)<br>+<br>English<br><br><u>Language exposure:</u><br>Spoke German and other language at home, and were using German and English at school | | | |
| **53**<br><br>**HT** | Poarch & Bialystok. (2015). Bilingualism as a model for multitasking. | Mean = 9;6 years | Monolinguals (English) | <u>Languages:</u><br>Bilinguals:<br>English<br>+<br>Bengali<br>Cantonese<br>Farsi<br>French<br>German<br>Gujarati<br>(+ 16 additional languages)<br><br>Trilinguals:<br>English<br>+<br>Arabic<br>Cantonese<br>French<br>Greek<br>Gujarati<br>Hindi<br>(+ 14 additional languages) | Executive function (EF):<br>- Inhibition | Modified Flanker task with 4 trial types:<br>- Baseline trial: no flankers<br>- Neutral trial: chevron flanked by diamonds<br>- Congruent trial: chevron flanked by same-direction chevrons<br>- Incongruent trial: chevron flanked by opposite-direction chevrons | Bilinguals and trilinguals were faster on incongruent trials than monolinguals ($ps$<.02); bilinguals and trilinguals did not significantly differ from one another. |

| | | | | | | | |
|---|---|---|---|---|---|---|---|
| | | | | <u>Language exposure:</u><br>Bilinguals: Used English in school/community and other language at home; parental report indicated roughly balanced language use<br><br>Trilinguals: Same as bilinguals but with 2 different home languages | | | |
| **54**<br><br>**HM** | Poarch & van Hell. (2012). Executive functions and inhibitory control in multilingual children: Evidence from second language learners, bilinguals, and trilinguals. *<br><br>***Trilinguals are not discussed due to lack of language background information | **<u>Experiment 1</u>:**<br><br>5-8 years<br><br>**<u>Experiment 2</u>:**<br><br>Same children 6-8 months later | **<u>Experiment 1</u>:**<br><br>Monolinguals (German)<br><br>**<u>Experiment 2</u>:**<br><br>German second-language learners (SLLs) of English | **<u>Experiment 1:</u>**<br><br><u>Languages:</u><br>German<br>+<br>English<br><br><u>Language exposure:</u><br>Used both languages at home and in school<br><br>**<u>Experiment 2:</u>**<br><br>Same group as Experiment 1 | **<u>Experiment 1</u>:**<br><br>Executive function (EF):<br>- Attentional control<br><br>**<u>Experiment 2</u>:**<br><br>Executive function (EF):<br>- Attentional control | **<u>Experiment 1</u>:**<br><br>Simon task<br><br>**<u>Experiment 2</u>:**<br><br>Attentional Networks Task (ANT) | **<u>Experiment 1</u>:**<br><br>No significant differences emerged between monolinguals and bilinguals (*p*=.062).<br><br>**<u>Experiment 2</u>:**<br><br>Overall performance in terms of RT and Errors did not differ between groups.<br><br><u>Alerting</u>: no significant difference emerged between groups.<br><br><u>Orienting</u>: Bilinguals showed greater effect than SLL's (*p*=.042).<br><br><u>Executive</u>: SLLs less efficient than bilinguals (*p*=.019). |
| **55**<br><br>**HM** | Struys, Duyck, & Woumans. (2018). The role of cognitive development and strategic task tendencies in the bilingual advantage controversy. | Younger group: 6 years<br><br>Older group: 11 years | Monolinguals (Dutch or French) | <u>Languages:</u><br>Dutch<br>+<br>French<br><br><u>Language exposure:</u><br>High proficiency in both languages (M prof. score = 3-3.5/4) | Cognitive control (CC) | Simon task<br><br>Flanker task | <u>Simon task</u>:<br>Young monolinguals showed a smaller Simon effect; no differences emerged between older groups.<br><br><u>Flanker task</u>:<br>A smaller Flanker effect emerged for older bilinguals than monolinguals.<br><br>Monolinguals had higher accuracy scores. |
| **56**<br><br>**HM** | Tse & Altarriba. (2014). The relationship between language | 5-9 years | Low-proficiency bilinguals | <u>Languages:</u><br>Cantonese<br>+<br>English | Executive function (EF):<br>- Task switching<br>- Attentional control<br><br>Working memory (WM) | <u>EF:</u><br>Simon task<br><br>Simon switching task | EF:<br><u>Simon task</u>: High L1/L2 proficiency predicted faster RTs.<br><br>Higher L2 proficiency predicted Simon |

| | | | | | | | |
|---|---|---|---|---|---|---|---|
| | proficiency and attentional control in Cantonese-English bilingual children: evidence from Simon, Simon switching, and working memory tasks. | | | Language exposure:<br>Tested participants on a continuum from low to high proficiency as measured by vocabulary tests | | WM:<br>Operation span task | effect.<br><br>Simon switching: None of the DVs was predicted by L2 proficiency; L1 proficiency predicted overall RT; neither L1 nor L2 proficiency predicted switch cost.<br><br>WM:<br>Bilinguals with higher L1/L2 proficiency outperformed lower proficiency subjects. |
| **57**<br><br>**HM** | Vivas, Chrysochoou, Ladas, Salvari. (2020). The moderating effect of bilingualism on lifespan cognitive development. | 5-13 years | Monolinguals (Greek) | Languages:<br>Greek<br>+<br>Albanian*<br><br>*Note: Older children in the group also had exposure to English in school.<br><br>Language exposure:<br>Spoke Albanian primarily in family context and Greek in educational and social settings | Executive function (EF):<br>- Attention network components (conflict/resistance to interference, alerting and orienting)<br>- Monitoring (indexed by overall RTs on ANT) | Attention Network Task (ANT) | Bilingualism significantly moderated the relationship between age and overall reaction times (RT; used as an index of monitoring ability) on the ANT, however correlation coefficients indicated that bilingual children actually showed a smaller improvement in RT with age than monolingual children.<br><br>Bilingualism did not significantly moderate the relationship between age and any of the ANT effects (conflict, orienting, or alerting). |
| | | | | **ADOLESCENT** | | | |
| **58**<br><br>**HM** | Gathercole, Thomas, Vinas Guasch, Kennedy, Prys, Young, Roberts, Hughes, & Jones. (2016). Teasing apart factors influencing executive function performance in bilinguals and monolinguals at different ages. | Children: 4-5 years<br><br>Adolescents: Mean = 14;9 years | Monolinguals (English) | Languages:<br>English<br>+<br>Welsh<br><br>Language exposure:<br>3 groups:<br>- Only English used at home (OEH)<br><br>- Only Welsh used at home (OWH)<br><br>- Welsh and English used at home (WEH) | Executive function (EF):<br>- Interference suppression/conflict resolution<br><br>General non-verbal cognitive ability | EF:<br>Simon task<br><br>General cognitive ability:<br>Children: McCarthy Scales of Children's Abilities<br><br>Adolescents: Raven's Progressive Matrices | EF:<br>Simon task - accuracy:<br>Bilingualism status was not a significant predictor for either age group in either Congruent or Incongruent trials.<br><br>Simon task - RT:<br>No significant predictors emerged for children.<br><br>For adolescents, Home Language was a significant predictor, with OWH adolescents having faster RTs in both conditions than monolinguals (but there was no effect of Bilingualism Status as a whole).<br><br>Simon task - Difference scores:<br>Neither Bilingualism Status nor Home Language was a significant predictor for |

| | | | | | | | |
|---|---|---|---|---|---|---|---|
| | | | | | | | accuracy or RT difference scores, for either age group<br><br>General cognitive ability:<br>No differences emerged between language groups. |
| **59**<br><br>**HM** | Kapa & Colombo. (2013). Attentional control in early and later bilingual children. | 5;8 – 14;11 years | Monolinguals (English) | Languages:<br>Spanish<br>+<br>English<br><br>Language exposure:<br>Spoke both languages daily; attended school in English<br><br>Split up by age of acquisition into Early (E-BIL) and Late Bilingual (L-BIL) groups | Attentional control | Child Attentional Network Task (ANT) | E-BIL children had faster RTs on ANT than the L-BIL and monolingual groups.<br><br>L-BIL group did not differ from monolingual group on RT.<br><br>Groups did not differ on ANT accuracy (suggesting higher efficiency on the task for E-BIL group).<br><br>There were no significant differences between groups for the 3 ANT networks. |
| **60**<br><br>**HM** | Krizman, Bradlow, Lam, & Kraus. (2017). How bilinguals listen in noise: Linguistic and non-linguistic factors. | Mean = 14.6 years | Monolinguals (English) | Languages:<br>Spanish<br>+<br>English<br><br>Language exposure:<br>Mean English = 58.4% daily<br>Mean Spanish = 41.6% daily | Word-in-noise perception<br><br>Tone-in-noise perception | Word task:<br>Words-In-Noise test<br><br>Tone tasks:<br>Backward masking<br><br>Simultaneous masking | Word in noise:<br>No significant differences emerged.<br><br>Tone in noise:<br>Bilinguals outperformed monolinguals on both tasks (*p*=.036; *p*=.033). |
